# Container buildings used for residential and business purposes in Johannesburg, South Africa and potential heat-related health risks

**DOI:** 10.12688/f1000research.138968.3

**Published:** 2024-10-14

**Authors:** Tanganedzeni Mfamadi, Kimberley Chivimbo, Philistase Mogadime, Shalin Bidassey-Manilal, Thandi Kapwata, Natasha Naidoo, Caradee Y Wright

**Affiliations:** 1 Department of Environmental Health, Faculty of Health Sciences, University of Johannesburg, Johannesburg, South Africa; 2 Department of Environmental Health, University of Johannesburg, Johannesburg, South Africa; 3 Environment and Health Research Council, South African Medical Research Council, Johannesburg, South Africa; 4 Environment and Health Research Council, South African Medical Research Council, Durban, South Africa; 5 Department of Geography, Geoinformatics and Meteorology, University of Pretoria, Pretoria, South Africa; 6 Environment and Health Research Council, South African Medical Research Council, Pretoria, South Africa

**Keywords:** Climate change, Environmental health, Temperature; Thermal comfort; Urban area

## Abstract

**Background:**

Outdoor and indoor air temperature affects human health and wellbeing. Climate change projections suggest that global temperatures will continue to increase, and this poses a threat to health. Buildings (for housing and business purposes) that can protect humans from the adverse effects of temperature are essential, especially in the context of climate change.

**Method:**

In this cross-sectional study, we measured the indoor temperature inside shipping containers comprising a seven-storey block of apartments and businesses in Johannesburg, South Africa for 14 days. We assessed indoor temperature and relative humidity; evaluated measured temperatures in relation to thresholds known to be associated with adverse health risks; and sought to understand heat-health perceptions and symptoms of people living and working in shipping container units.

**Results:**

Median indoor apparent temperature (AT) (a combination of temperature and relative humidity) was 16°C with values ranging from 6°C (observed at 8:00) to 42°C (observed at 17:00). Insulated units had temperatures between 2°C and 9°C cooler than the uninsulated unit. Heat-health risks from AT exposure were likely in all units, although there was variation in the number of occurrences that AT measurements exceeded the four symptom bands of caution, extreme caution, danger and extreme danger. Indoor AT was found to be 7°C higher on average when compared to outdoor AT. Some participants believed that their units were hot during hot weather and most people opened windows or did nothing during hot weather. Few participants reported experiencing adverse heat-health impacts, except for experiencing headaches (58%) and feeling tired or weak (40%).

**Conclusion:**

Residents, tenants, or business owners using shipping containers should consider insulation installation and adequate windows/air conditioning for ventilation, especially in hot climates. Further research and awareness regarding heat-health risks of living or working in these spaces is needed.

## Introduction

Properly designed and constructed residential, business and public service spaces can help prevent disease, reduce poverty, and increase quality of life. ^1^^,^^2^ Adequate, safe housing and working environments are essential in the current global context of urbanisation, ageing populations, and climate change. ^1^ In South Africa, around 80% of the population of around ~60 million people live in formal dwellings, 11% live in informal dwellings such as shacks, 4% live in traditional dwellings and the other 5% is unknown. ^3^ Government-provided, formal, low-cost housing has failed to address the growing informal housing problem in South Africa. ^4^ Recently, initiatives by developers have increased the use of shipping containers to provide for South Africans who earn very little. As low-cost housing solutions, several shipping containers have been combined into a single-storey or stacked to make a multi-storey block of apartments. To serve poorer communities, shipping containers have been used as clinics as well as “spaza shops” to sell essential items in townships. ^5^^,^^6^

In wealthier countries, shipping containers have been used as a sustainable housing option, sometimes called an ‘eco-pod’. ^7^ This form of housing has also been used during emergency relief after extreme weather events displace people from their homes, as was the case in the Philippines. ^8^ Similarly, following Hurricane Katrina in 2005, the Christchurch earthquake in 2011 and the Victoria (Australia) Black Saturday bushfires in 2009, shipping container dwellings were used to temporarily house displaced populations. ^9^

It is crucial, when repurposing shipping containers for residential or workspace purposes, to ensure that the temperature inside the container is kept stable, thermally appropriate, and conducive to human habitation. ^8^ Shipping containers are made of Corten steel, which possesses the physical properties that make it weldable and rust-resistant. ^10^ Although Corten steel can withstand different weather elements, it produces high thermal conductivity which enables it to absorb heat more efficiently transmitting it from the exterior of the building to the interior, which can make the interior extremely hot and uncomfortable for humans. ^10^ Temperature measurements made inside shipping containers while they were on ships have shown that temperatures may exceed 60°C. ^11^ Therefore, indoor temperatures in shipping containers may pose a challenge for their use as dwellings, especially in hot climates.

Elrayies ^12^ assessed the thermal performance of shipping container architecture in hot and humid Port Said, Egypt. The temperature within the uninsulated shipping container reached an excess of 44 °C in the afternoon, whilst insulation kept indoor conditions slightly cooler than outside (28 °C – 31 °C), depending on the type of insulation applied. Closed-cell spray polyurethane foam performed better than rock wool, wool, or straw. Nevertheless, even with the application of thermal insulation, the reported temperatures remain within the range that poses a high risk of potential health impacts (27 °C – 32 °C). ^13^ In the Philippines, different types of insulation such as foam and fibreglass batting did not improve the indoor thermal conditions of the shipping containers in a tropical (hot and humid) climate. ^8^ The marginal enhancement in thermal comfort achieved through the application of closed-cell spray polyurethane may be deemed questionable, especially in light of the associated expense.

The only study analysing temperatures inside shipping containers was of classrooms in Johannesburg, South Africa, which showed temperatures in excess of 40°C during the summer. ^14^ There have been no studies to characterise thermal comfort conditions by measuring both temperature and humidity inside shipping containers converted into residences or businesses in South Africa. Therefore, we aimed to measure indoor temperature and humidity inside shipping containers comprising a seven-storey block of apartments and businesses in Johannesburg, South Africa. There were three study objectives: 1) to assess temperature inside shipping container units used as dwellings and places of business; 2) to evaluate measured temperatures in relation to thresholds known to be associated with adverse health risks; and 3) to understand heat-health perceptions and symptoms of people living and working in shipping container units. The study data may form a foundation for the development of guidelines and regulations required to improve the habitability of converted containers. In addition, these findings are important to inform policymaking and health awareness campaigns related to living and working in shipping containers in hot climates.

## Methods

### Study design

**Figure 1.**
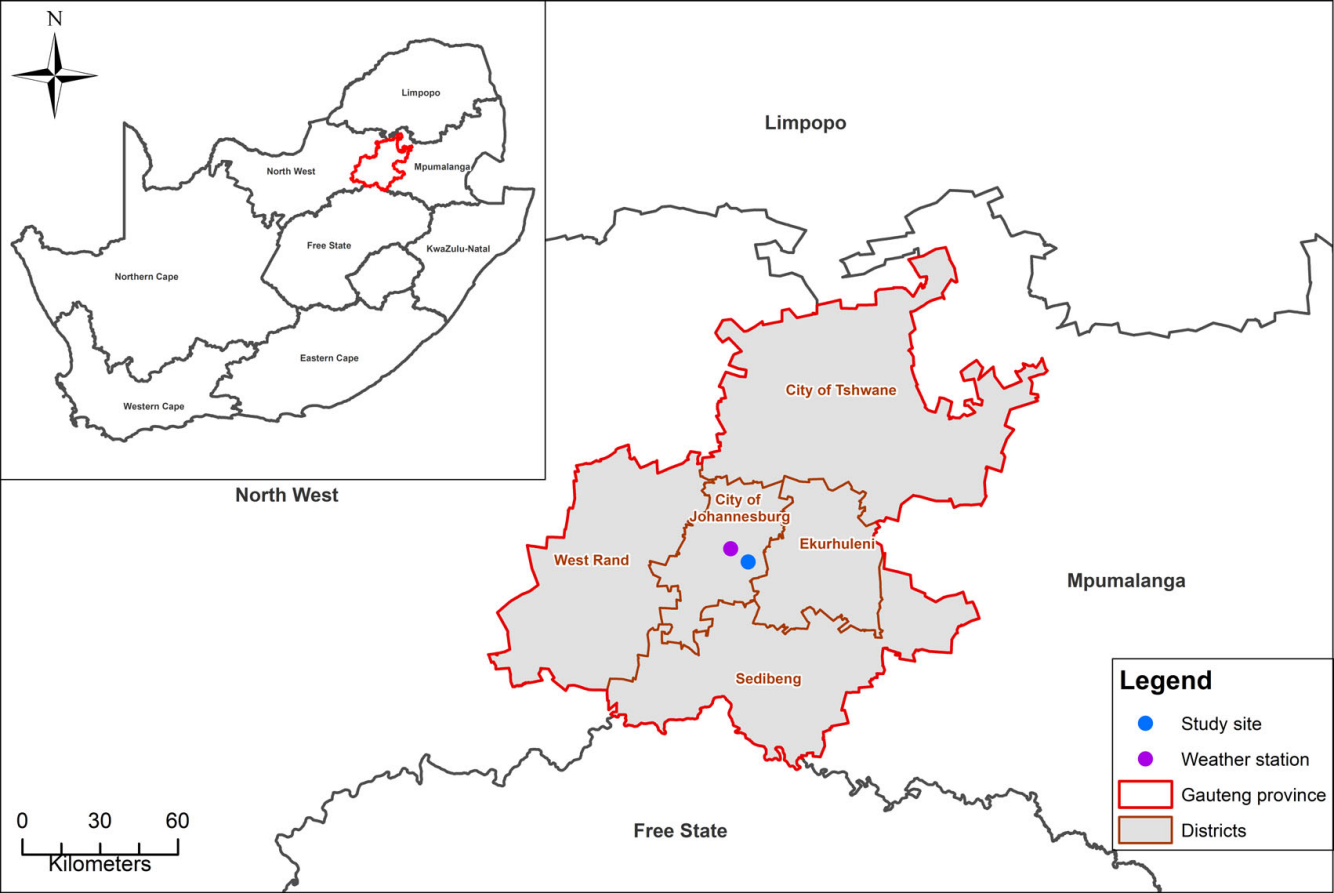
Map showing the study site, Maboneng (blue dot) and the weather station (purple dot).

**Figure 2.**
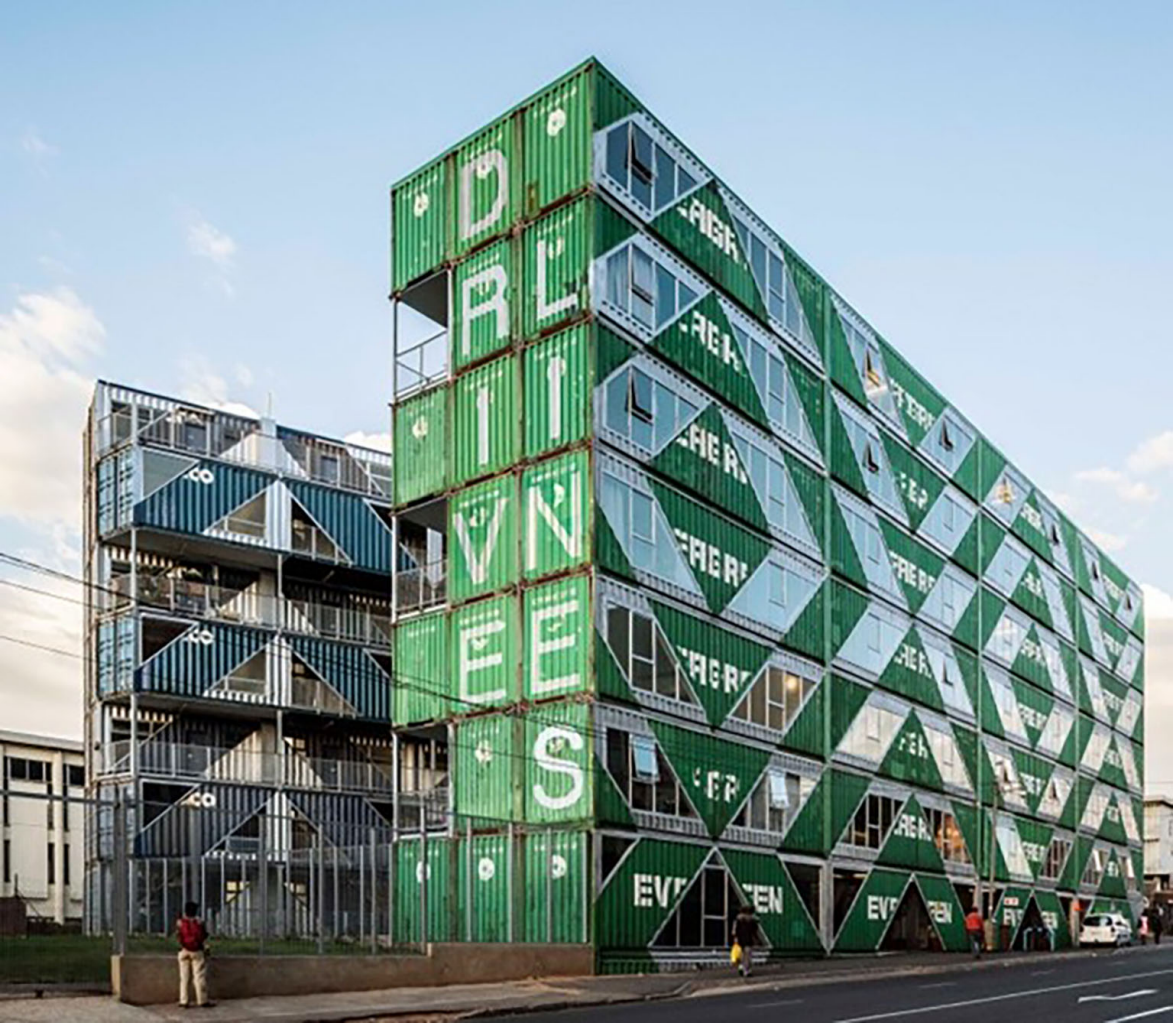
Street view of the container building comprising businesses and residences. (Photo credit: Driveline Studios).

This cross-sectional study was conducted in the suburb of Maboneng ( Figure 1), located in the City of Johannesburg, South Africa. Strengthening the Reporting of Observational Studies in Epidemiology (STROBE) guidelines were used to plan and execute this study. The STROBE guidelines ensure the highest quality of observational study data and consist of 22 checklist items that should be addressed in a study. ^15^ Maboneng, a mixed-use neighbourhood within the central business district of the city. The focus of our study was a building made of 140 shipping containers ( Figure 2) repurposed into 107 units that are either residential apartments (n=103) or businesses (n=4). All shipping containers were painted in dark colours that are either dark blue or dark green. We aimed to install temperature loggers in ten units from the 12 September to 25 September 2021 after obtaining permission from the owner of the building and the tenants/owners of the units. The sampling took place in September because it is spring time in South Africa, and high temperatures were recorded in previous years during this month.

### Ethical approval

Research ethics clearance was obtained from the University of Johannesburg Faculty of Health Sciences Research Ethics Committee (REC-1055-2021, 4 June 2021).

### Informed consent

Written informed consent was obtained from all participants for participation in the study as well as publication of their self-reported data.

### Meteorological measurements

Temperature loggers called iButtons were installed in the container units to measure indoor temperature. Units 1 to 8 had similar interior characteristics. The units contain a built-in cupboard and closet, a stove, a fridge, TV stand, couch/chairs and a bed. Unit 9, which was the salon had a wall-to-wall table with 7 chairs, a cabinet where they store their tools, and some tools were, displayed on the table. The office contained a table, four chairs and a small free-standing cabinet. iButtons are small, portable sensors that record temperature and relative humidity onboard for download after monitoring. iButtons were uniformly installed against a cupboard, away from the walls, in all ten containers from the 12 September to 25 September 2021. This approach was made instead of suspending the iButton from the ceiling to minimise intrusion of the instrument to the inhabitant and has been done successfully before. ^16^ Data were downloaded as.txt files and imported into R software ^17^ for analysis.

Outdoor temperature and relative humidity data for the meteorological station closest to the container building were obtained from the South African Weather Service (SAWS). Wind data were also available. Meteorological data within the study period (12 September to 25 September 2021) were provided at 10-minute intervals which were converted to hourly interval calculations. Unfortunately, 50% of the data were missing.

### Apparent temperature calculations and heat-health risks

Indoor and outdoor temperature and relative humidity data were used to calculate indoor and outdoor apparent temperature (AT), respectively. AT is a calculation that considers ‘real-feel’ temperature and is an indicator of thermal comfort as well as gives an indication of potential heat-health impacts based on defined thresholds. ^13^

AT was calculated as follows:

AT=Ta+0.33xe–0.70xws–4.00 (1)

Where:

Ta = dry bulb temperature (°C)

e = water vapour pressure (hPa)

ws = wind speed (set to 0 for indoor conditions, and using the SAWS wind speed data for outdoors)

Water vapour was calculated using Equation 2:

$$E=\mathrm{RH}/100\;\times \;6.105\;\times \;\exp\;(17.27\;\times \;\mathrm{Ta}/\left(237.7+\mathrm{Ta}\right)$$ (2)

AT is given in degrees Celsius (°C) and was calculated for hourly intervals.

AT measurements made inside the shipping container units were considered in relation to thresholds known to be associated with adverse health impacts ( Table 1).

**Table 1.** Apparent temperature (AT) thresholds and potential health impacts. ^11^

Symptom band	Classification	AT range (°C)	Classified “Effect on Body”
I	Caution	27–32	Fatigue possible with prolonged exposure and/or physical activity
II	Extreme caution	33–39	Heat stroke, heat cramps, or heat exhaustion possible with prolonged exposure and/or physical activity
III	Danger	40–51	Heat cramps or heat exhaustion likely, and heat stroke possible with prolonged exposure and/or physical activity
IV	Extreme Danger	>51	Heat stroke highly likely

### Survey questionnaire

We administered a short survey questionnaire to occupants of the units after obtaining their informed consent. The questionnaire was used to gather information from randomly selected participants (simple random sampling with equal chance of selection) about demographic and socio-economic characteristics, heat-related symptoms ever experienced while occupying the container units, perceptions around heat, and container characteristics. Participants were eligible for the study when they lived in an apartment in the container building or worked in one of the businesses in the container building; they were 18 years old or older; and they consented to participate using an informed consent process.

Sample size was determined by the number of fieldworkers (n=3) and the number of fieldwork days available for the study (n=14) together with the required time to conduct the survey (~20 minutes). Some of the participants were not conversant in English so the researchers communicated in other languages such as Zulu, Sotho and Tshwane during data collection. Responses were anonymised and coded prior to analysis in STATA (version 16) (StataCorp, 2019). ^18^

A total of 62 participants (58%) answered the survey. Other residents and business owners (n=45) in the building were not available to participate during the study period.

### Statistical analyses

Descriptive statistics were used to summarize participant characteristics, diurnal and daily patterns of indoor AT and outdoor AT in the shipping containers. Missing data were left as is and no data were inferred. ^17^ Data were analysed using R, a language and environment for statistical computing. No sensitivity analyses were done.

## Results

### Apparent temperature findings

**Figure 3.**
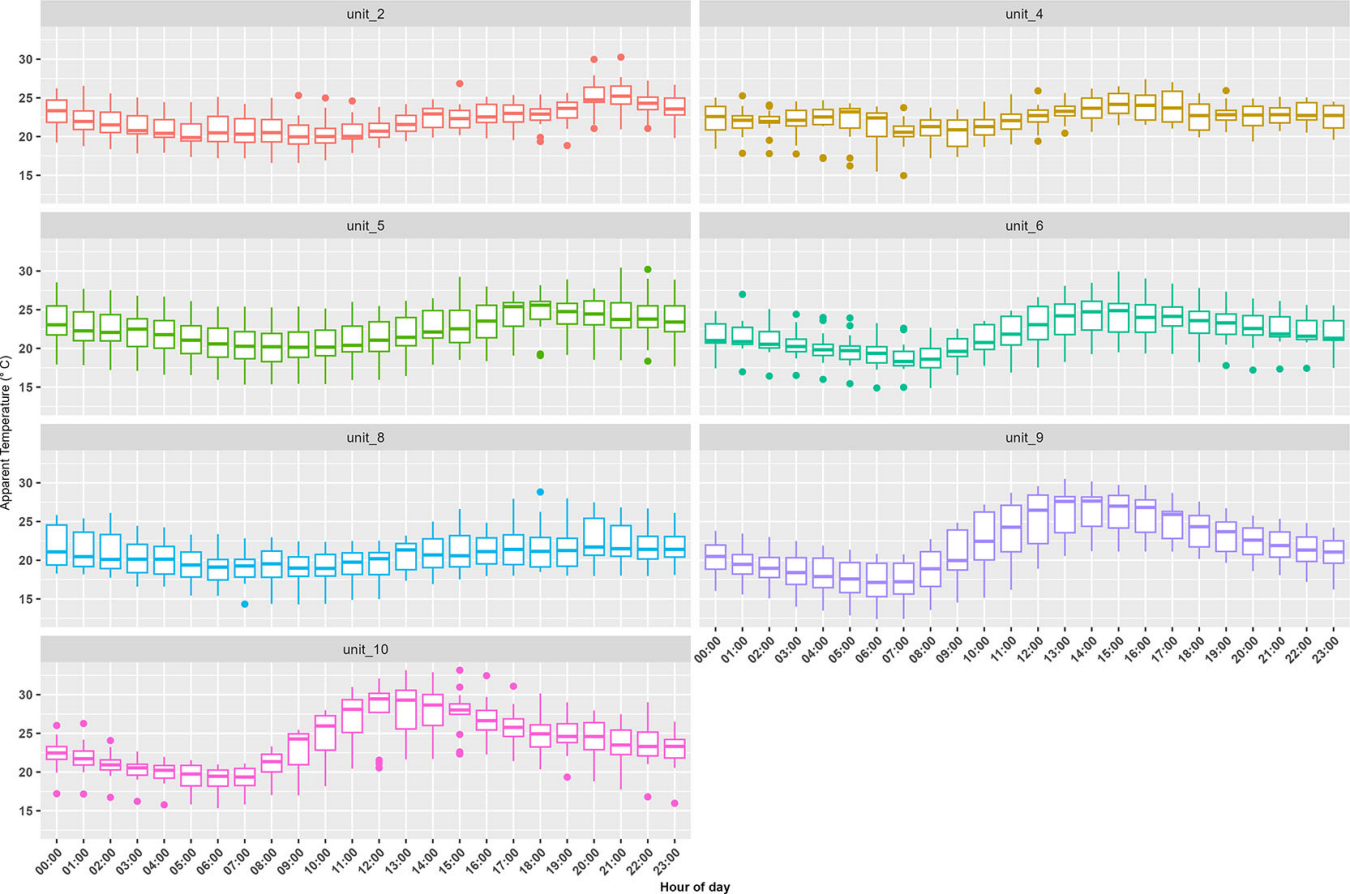
Trends in hourly apparent temperature (°C) indoor measurements made inside the container building’s apartments and businesses during the study period from 12 September to 25 September 2021. The whiskers indicate the 95 ^th^ and 5 ^th^ percentile, the box indicates the interquartile range, and the middle line is the median.

The study took place during the austral spring (i.e., September) when average outdoor temperatures in Johannesburg are ~8°C (minimum) and ~24°C (maximum). ^19^ We successfully measured temperature and relative humidity in seven container units (three of the iButtons failed during the study campaign). Indoor AT measurements made in the container units displayed similar diurnal patterns with the warmest temperatures occurring in the mid-afternoon ( Figure 3). Median indoor AT was 22°C with values ranging from 12°C (observed at 6:00 in unit 9) to 33°C (observed at 15:00 in unit 10). For outdoor conditions, the median AT was 14°C with values ranging between 6°C to 23°C.

**Figure 4.**
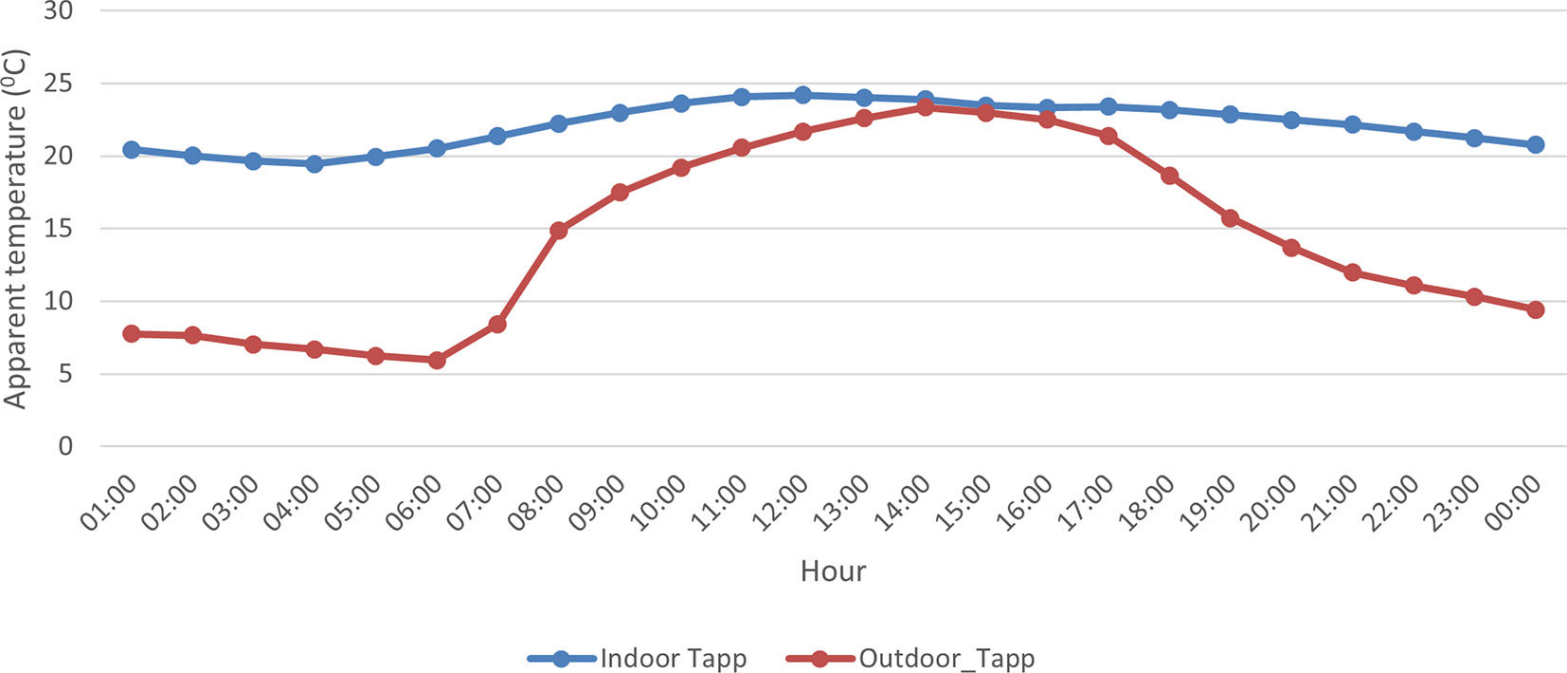
Average trends in hourly interior apparent temperatures (blue line) with the average applied both overall containers and over all days and outdoor (red line) during the study period from 12 September to 25 September 2021.

A comparison of indoor and outdoor AT showed that indoor AT was ~ 7°C higher on average compared to outdoor AT during the study campaign ( Figure 4). However, indoor and outdoor conditions were very similar in the afternoon between 13:00 and 17:00 with an AT difference of ~ 1°C. Also, outdoor AT showed greater variability compared to indoor AT with steep increases observed in the morning and sharp declines in the afternoon.

**Figure 5.**
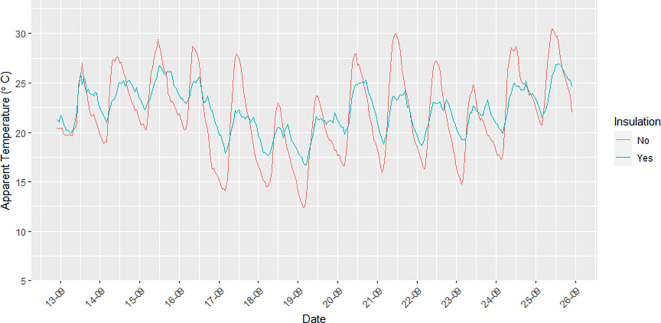
Diurnal trends in indoor AT (°C) in a container apartment without insulation (red line) and with insulation (blue line) during the study period from 12 September to 25 September 2021.

Units 2 (residential), 4 (residential), 5 (residential), 6 (residential), 8 (residential), and 10 (business) had insulation installed in the container walls and roof in an attempt to moderate the impacts of outdoor temperature on indoor temperature. Unit 9 (business) did not have insulation. Figure 5 illustrates the difference that insulation makes in relation to AT recorded inside the container units with and without insulation. The amplitude of the AT measurements made inside insulated units was smaller than that seen for the non-insulated units for both minimum and maximum indoor AT. The difference in AT for insulated versus non-insulated units ranged between ~2–9°C.

Heat-health risks from AT exposure occurred in all units although there was variation in the number of occurrences in AT measurements exceeded the four symptom bands ( Table 2). AT in unit 5 reached the ‘danger’ threshold of between 40 to 51°C once during the study period. In unit 10 there were 13 hourly exceedances of the ‘extreme caution’ threshold of 33–39°C. All units in which AT was measured experienced some exceedances of the ‘caution’ threshold with the greatest occurrences taking place in units 9 (uninsulated) and 10 (insulated).

**Table 2.** Mean (hourly range) AT measurements made in each of the seven units and the number of occurrences (hourly) when AT levels exceeded the four heat-health symptom bands.

Unit number in the study (type, storey)	AT mean (hourly range)	Frequency of hourly occurrences n (%)			
		Caution 27–32°C	Extreme caution 33–39°C	Danger 40–51°C	Extreme danger >51°C
2 (residential, 2 ^nd^)	6–30°C	22 (100)	-	-	-
4 (residential, 4 ^th^)	6–28°C	8 (100)	-	-	-
5 (residential, 4 ^th^)	6–42°C	28 (90)	2 (6)	1 (3)	
6 (residential, 5 ^th^)	6–30°C	22 (100)	-	-	-
8 (residential, 6 ^th^)	6–28°C	7 (100)	-	-	-
9 (business, ground)	6–32°C	62 (98)	1 (1)	-	-
10 (business, ground)	6–34°C	80 (86)	13 (13)	-	-

### Questionnaire findings

The majority of the participants were older than 26 years of age, were male and had completed high school ( Table 3). At the time of the survey, the shipping container structure was 5 years old, having been assembled in 2017, when units were available to rent or purchase. All participants of this study were utilizing the containers for less than 6 months. All surveyed participants reported that their units had windows and were occupied by one or more people.

**Table 3.** Descriptive characteristics of the study participants who lived in the residential apartments (N=62).

Variable	Frequency (n)	Frequency (%)
**Age (years):**		
**Younger than 18**	0	0
**18 – 25**	25	40
**26 – 35**	33	53
**36 – 45**	4	6
***Missing***	*0*	*0*
**Gender:**		
**Male**	41	66
**Female**	21	33
***Missing***	*0*	*0*
**Highest level of education:**		
**No formal education**	0	0
**Primary school completed**	0	0
**Attended high school**	2	3
**Completed high school**	11	17
**Attended university**	19	30
**Completed university**	30	48
***Missing***	*0*	*0*
**Duration living/working in container:**		
**Less than a month**	3	4
**1 month**	1	1
**3 months**	11	17
**6 months**	14	22
**More than 6 months**	33	53
***Missing***	*0*	*0*
**Number of occupants in unit:**		
**1**	37	59
**2-3**	21	33
**3-5**	4	6
**>5**	0	0
***Missing***	*0*	*0*

All participants reported that their container units had windows and two-thirds said that these windows, when opened, helped to cool down their units ( Table 4). Findings were ambivalent regarding whether participants believed that their units were hot during hot weather and most people opened windows or did nothing during hot weather. Few participants reported experiencing adverse heat-health impacts, except for experiencing headaches (58%) and feeling tired or weak (40%) ( Table 5).

**Table 4.** Participants’ perceptions related to heat and heat-behaviours (N=62).

Variables	Frequency (n)	Frequency (%)
**Are there windows in the unit?**		
**Yes**	62	100
**No**	0	0
***Missing***	*0*	*0*
**Do the windows help to cool down the unit?**		
**Yes**	59	95
**No**	3	5
***Missing***	*0*	*0*
**Do you think the temperatures inside the containers are comfortable during hot weather conditions?**		
**Yes**	33	54
**No**	29	46
***Missing***	*0*	*0*
**What do you use to keep your unit cool on hot days?** *		
**An air conditioner**	2	3
**An electric fan**	11	17
**Open windows**	20	32
**Nothing**	29	46

* Participants could select multiple responses.

**Table 5.** Heat-related symptoms were reported by the study participants (N=62).

Self-reported heat-related symptoms	Frequency (n)	Frequency (%)
**Headache**		
**Yes**	36	58
**No**	26	42
***Missing***	*0*	*0*
**Dizziness**		
**Yes**	6	9
**No**	56	91
***Missing***	*0*	*0*
**Nausea or vomiting**		
**Yes**	9	15
**No**	53	85
***Missing***	*0*	*0*
**Heat cramps**		
**Yes**	4	6
**No**	58	94
***Missing***	*0*	*0*
**Heat stroke**		
**Yes**	1	2
**No**	61	98
***Missing***	*0*	*0*
**Heat rash**		
**Yes**	10	16
**No**	52	84
***Missing***	*0*	*0*
**Losing consciousness (fainting)**		
**Yes**	1	2
**No**	61	98
***Missing***	*0*	*0*
**Tiredness or weakness**		
**Yes**	25	40
**No**	37	60
***Missing***	*0*	*0*
**Excessive sweating**		
**Yes**	14	23
**No**	48	77
***Missing***	*0*	*0*
**Confusion**		
**Yes**	4	6
**No**	58	94
***Missing***	*0*	*0*
**Low concentration**		
**Yes**	10	16
**No**	52	84
***Missing***	*0*	*0*

## Discussion

This study has successfully clarified the low indoor thermal comfort characteristics of shipping container units used for living and business purposes in a hot and humid environment in the metropolitan city of Johannesburg. Indoor and outdoor temperatures followed a similar diurnal pattern with far less variation for indoor conditions compared to outdoors. Indoor temperatures were consistently warmer than outdoor temperatures (~ 7°C higher on average) and with some non-optimal high temperature exceedances presenting a reason for concern regarding people’s heat-health risks. Laksitoadi and As Syarif ^20^ measured temperatures inside shipping containers repurposed into offices in Badung, Indonesia which has a hot, wet climate. In contrast to our findings, this study found that indoor temperatures were on average cooler than outdoor temperatures. Similar to our findings, the location of the offices in the building by floor did not influence indoor temperatures. There were no consistent patterns in indoor temperatures by storey/level.

Insulation of shipping container units may not be adequate for the maintenance of thermal comfort conditions to prevent heat-health risks. All units in which measurements were made had exceeded the heat-health risk threshold values related to ‘caution.’ Three units exceeded the threshold values related to ‘extreme caution’ of 33–39°C. One unit exceeded the threshold values related to ‘danger’ of 40–51°C. Two container units, one insulated and the other not had the highest frequency of temperature occurrences surpassing the ‘caution’ (27–32°C) threshold. These temperatures are associated with fatigue which is equivalent to prolonged heat exposure and/or physical activity. This suggests that an alternative means to ensure thermal comfort inside shipping container residential and business units is required. Suggested solutions include green/vegetated roofs and outside walls and double-glazing on windows. ^21^ Solar-powered air-conditioning may also be an alternative energy source for indoor cooling, especially in energy-constrained environments such as South Africa. Window shading with awnings or blinds may also help maintain cool indoor temperatures and the application of light-colored exterior paint can reduce interior temperatures.

Several factors influence indoor temperature in dwellings such as the size of the dwelling, number of doors and windows, shading and human behaviour in relation to ventilation and heating/cooling. ^22^ Window shading with awnings or blinds may also help maintain cool indoor temperatures and the application of light-colored exterior paint can reduce interior temperatures. Shipping containers are a standard size (~14 m ^2^ to 30 m ^2^) – in this apartment block, the open plan studio units were made from containers varying between 28–56 m ^2^ and each had an open landing outdoor space on the ‘inside-facing’ sides of the building. These spaces were less likely to receive natural wind-driven ventilation due to the nature of the balcony location between the two ‘wings’ of the building above the central courtyard (see Figure 2). It will be important for future buildings to consider ventilation and through breezes in their design and construction to ensure indoor temperatures may be reduced, especially in hot climates.

Study participants were ambivalent regarding whether their units were hot during hot weather; this may be since they had only lived in the unit for around 6 months and had yet to experience the heat of summer between December to February. Few people reported adverse heat-health impacts except for experiencing headaches and feeling tired/weak. Several symptoms and health effects are associated with exposure to non-optimally high indoor temperatures. High indoor temperature exposure has been associated with respiratory, cardiovascular, and mental health effects. ^23^ While our study participants were relatively young, they were also vulnerable to the health impacts of extreme heat. A recent study found stronger associations between days of extreme heat and a higher risk of emergency department visits for any cause, heat-related illness, renal disease, and mental disorders among young and middle-aged compared to older adults. ^24^

While shipping containers are perceived to be a good alternative for building dwellings, businesses, school classrooms, relief shelters etc. due to their relatively low cost, ease of availability, speed to completion during the build, low maintenance, weather-resistant, supposedly relatively low environmental impact (low carbon footprint since at end-of-life at sea/on the road, it is repurposed/upscaled), they may release toxic substances if they had a history of carrying chemicals and they need insulation since the metal frames do not possess insulating properties. The latter is particularly important, especially in light of global warming. South Africa is projected to experience an increase in average outdoor temperature between 4–6°C by 2100. ^25^ Furthermore, a heat stress assessment study found that the central business district within of the city of Johannesburg, which is where the containers were located, exhibited urban heat island characteristics due to high building densities and sparse vegetation. ^26^ Thus, residents are at increased risk of heat stress because of exposure to high night and daytime temperatures. Simulations of future local climates estimated increases of up to 5°C, therefore efficient means of maintaining indoor thermal comfort are necessary. ^25^^,^^27^ When indoor temperatures inside shipping container units used for living and working mimic outdoor temperatures, and outdoor temperatures are likely to increase, there is reason for concern regarding the health and wellbeing of the occupants. Housing is a constitutional right in South Africa; however, the thermal comfort of the housing requires attention given future climate risks.

There were several study limitations. We only had ten iButtons available to us which limited our sample size and some of the iButtons failed so data were lost. In the future, two iButtons should be placed side-by-side to have a back-up device in the event that one logger fails to prevent data loss and to increase the accuracy and variation between the readings. Most of the participants mentioned that they would be out of the city for the holidays so that limited our sample period and sample size. The study period was relatively short (i.e., 14 days) and took place in spring; additional measurements should be made in summer when outdoor temperatures are likely higher compared to temperatures in spring. We should have asked business owners working in the business units about the nature of their business to understand whether indoor temperatures were affected by business activities, e.g., the use of hair dryers in a hair salon. We should have also recorded whether the units were north- or south-facing. It would be useful in future studies to conduct physiological measurements of inhabitants of container homes to assess possible heat-health related impacts during hot weather. One may also consider having the participants wear temperature-logging wearables to estimate personal exposure while living and/or working in container-made buildings.

Given the projected extreme outdoor temperatures (both heat and cold) due to climate change, it is imperative to devise strategies to enhance indoor thermal comfort. This is particularly important for low- and middle-income countries, ensuring the well-being of those forced to consider cheaper building options. The information gleaned from this study can be used to conceptualise future research to solve the thermal comfort limitations of shipping containers. Characterising measures to ensure thermal comfort inside shipping container residential and business units is warranted. Solutions such as green or vegetated roofs, exterior wall insulation options, double-glazing on windows, ^21^ and solar-powered air-conditioning could be further explored and optimized. Building on insights from similar studies in different climates, researchers can delve deeper into the performance of various insulation materials, considering factors such as cost-effectiveness and sustainability. The influence of building design is especially interesting, particularly ventilation through breezes and the direction of buildings. Indoor temperatures should be a focal point for architects and urban planners aiming to create climate-resilient structures. Future research could also extend beyond temperature measurements to include physiological assessments of inhabitants for personalized exposure estimates, providing a more holistic understanding of heat-health impacts. Finally, addressing study limitations by increasing sample size, incorporating backup devices, and extending data collection periods to both summer and winter would strengthen the reliability and applicability of future research in this domain.

## Conclusion

Housing characteristics influence indoor temperature, and this has an impact on human health and wellbeing. Shipping containers upscaled into living and working units in a modular apartment block is a viable alternative to low-cost housing. However, it is important to consider and factor into the building design indoor temperature stability and by inference, thermal comfort. Based on the results obtained from this study, it can be concluded that occupants of Drivelines studios are at risk of experiencing adverse heat-related symptoms as the apparent temperature calculations exceeded the four symptoms band of caution, extreme caution, danger and extreme danger. These symptoms are associated with fatigue, heat cramps, heat exhaustion and the possibility of a heat stroke. If the container-based buildings are adequately insulated, then temperatures inside these buildings can be controlled. The findings of future studies that analyse optimisation of thermal comfort in shipping containers should be used to advocate for and educate people who oversee the adaptation of shipping containers for either residential or business uses. Given the potential health symptoms and threats associated with non-optimal indoor temperatures, interventions and solutions for thermal comfort must be considered when building residential and business spaces with shipping containers in Africa and elsewhere around the world.

## Data Availability

Zenodo: iButton and Questionnaire data for container building in Johannesburg, https://doi.org/10.5281/zenodo.8085438. ^28^ This project contains the following underlying data: • Questionnaire data.xlsx (Questionnaire data) • Temperatures.csv (Temperature data from measurements made in the container rooms) Data are available under the terms of the Creative Commons Attribution 4.0 International license (CC-BY 4.0). Zenodo: STROBE checklist for **‘**Container buildings used for residential and business purposes in Johannesburg, South Africa and potential heat-related health risks’, https://doi.org/10.5281/zenodo.8143086. ^29^ Data are available under the terms of the Creative Commons Attribution 4.0 International license (CC-BY 4.0).

## References

[1] World Health Organization : WHO Housing and Health Guidelines. 23 November2018. Accessed 26 June 2023. Reference Source

[2] Kilian A : Shipping containers capturing imagination as alternative building blocks. Creamer Media’s Engineering News.12 February2016. Accessed 16 January 2024. https://www.engineeringnews.co.za/article/shipping-containers-capturing-the-imagination-as-alternative-building-blocks-2016-02-12

[3] Statistics South Africa: General Household Survey .2021. 23 June 2022. Accessed 26 June 2023. Reference Source

[4] Marutlulle NK : A critical analysis of housing inadequacy in South Africa and its ramifications. *Africa’s Public Service Delivery and Performance Review.* 2021;9(1). 10.4102/apsdpr.v9i1.372

[5] Sutherland M Krige K : Unjani “clinics in a container”: social franchising in South Africa. Emerald Insight. 2017;7(1):1–23. 10.1108/EEMCS-06-2016-0151

[6] Chipunza C Phalatsi BC : The influence of selected demographic factors on the choice of marketing communication tools: Comparison of foreign and local spaza shop owners in South Africa. *Acta Commercii.* 2019;19(1). 10.4102/ac.v19i1.752

[7] Lakot Alemdag E Aydin O : A study of shipping containers as a living space in context of sustainability. *ARTiUM.* 2015;3(1):17–29. Reference Source

[8] Zafra RG Mayo JRM Villareal PJM : Structural and thermal performance assessment of shipping container as post-disaster housing in tropical climates. *Civil Engineer J.* 2021;7(8):1437–1458. 10.28991/cej-2021-03091735

[9] Zhang G Setunge S Elmpt S van : Using shipping containers to provide temporary housing in post-disaster recovery: social case studies. *Procedia Economic Fin.* 2014;18:618–625. 10.1016/S2212-5671(14)00983-6

[10] Bob C : What Are Shipping Containers Made Of? Mobile Modular Solutions.Accessed 9 January 2024. https://www.mobilemodularcontainers.com/blog/what-are-shipping-containers-made-of

[11] TheShipsAI : How hot do shipping containers get? Accessed 26 June 2023. Reference Source

[12] Elrayies GM : Thermal performance assessment of shipping container architecture in hot and human climates. *Int. J. Adv. Sci. Engin. Inform. Tech.* 2017;7:1114–1126. 10.18517/ijaseit.7.4.2235 Reference Source

[13] Steadman RG : Norms of apparent temperature in Australia. Accessed 23 June 2023. Reference Source

[14] Bidassey-Manilal S Wright CY Engelbrecht JC : Students’ Perceived Heat-Health Symptoms Increased with Warmer Classroom Temperatures. *Int. J. Environ. Res. Public Health.* 2016;13(6):566. 10.3390/ijerph13060566 27338423 PMC4924023

[15] Cuschieri S : *The STROBE guidelines. Saudi J. Anaesth.* 2019;13(1). 10.4103/sja.SJA_543_18 PMC6398292 30930717

[16] Naicker N Teare J Balakrishna Y : Indoor Temperatures in Low Cost Housing in Johannesburg, South Africa. *Int. J. Environ. Res. Public Health.* 2017;14(11):1410. 10.3390/ijerph14111410 29156558 PMC5708049

[17] R Core Team: *R: A language and environment for statistical computing* *.* Vienna, Austria: R Foundation for Statistical Computing;2022. Accessed 24 June 2023. Reference Source

[18] StataCorp: *Stata Statistical Software: release 16* *.* College Station, TX: StataCorp LLC.;2019. Accessed 25 June 2023. Reference Source

[19] Climate Data: Johannesburg Climate (South Africa). 2023. Accessed 23 June 2023. Reference Source

[20] Latsitoadi B As Syarif MH : Refurbished shipping containers as architectural module in Bandung. *Advances in Social Science, Education and Humanities Research, Proceedings of the 3 ^rd^ International Conference on Dwelling Form (IDWELL 2020)* *.* 2020;475:1–9. 10.2291/assehr.k.201009.001

[21] Taleb H Elsebaei M El-Attar M : Enhancing the sustainability of shipping container homes in a hot arid region: A case study of Aswan in Egypt. *Architect. Engineer Design Manage.* 2019;15:459–474. 10.1080/17452007.2019.1628002

[22] Teare J Mathee A Naicker N : Dwelling Characteristics Influence Indoor Temperature and May Pose Health Threats in LMICs. Ann. Glob. Health. 2020 Aug 3;86(1):91. 10.5334/aogh.2938 32832385 PMC7413138

[23] Tham S Thompson R Landeg O : Indoor temperature and health: a global systematic review. *Public Health.* 2020Feb;179:9–17. 10.1016/j.puhe.2019.09.005 31707154

[24] Sun S Weinberger KR Nori-Sarma A : Ambient heat and risks of emergency department visits among adults in the United States: time stratified case crossover study. *BMJ.* 2021;375: e065653. 10.1136/bmj-2021-065653 34819309 PMC9397126

[25] Engelbrecht F Adegoke J Bopape M-J : Projects of rapidly rising surface temperatures over Africa under low mitigation. *Environ. Res. Lett.* 2015;10:085004. 10.1088/1748-9326/10/8/085004

[26] Hardy C Nel A : Data and techniques for studying the urban heat island effect in Johannesburg. Int. Arch. Photogramm. Remote Sens. Spatial Inf. Sci. 2015;XL-7/W3:203–206. 10.5194/isprsarchives-XL-7-W3-203-2015

[27] Souverijins N De Ridder K Veldeman N : Urban heat in Johannesburg and Ekurhuleni, South Africa: A meter-scale assessment and vulnerability analysis. *Urban Clim.* 2022;46:101331. 10.1016/j.uclim.2022.101331 36482986 PMC9720904

[28] Wright C : IButton and Questionnaire data for container building in Johannesburg.[Dataset]. *Zenodo.* 2023. 10.5281/zenodo.8085438

[29] Wright C : STROBE checklist for ‘Container buildings used for residential and business purposes in Johannesburg, South Africa and potential heat-related health risks’. *Zenodo.* 2023. 10.5281/zenodo.8143086 PMC11322856 39144543

