# Supplementary material for: Container buildings used for residential and business purposes in Johannesburg, South Africa and potential heat-related health risks
Source: F1000Res. 2024 Oct 14;12:929. Originally published 2023 Aug 3. [Version 3] doi: 10.12688/f1000research.138968.3 (PMC11322856; doi:10.12688/f1000research.138968.3)
Supplement: Peer review history

# Reviewer report: Reviewer response for version 3

**DOI:** 10.5256/f1000research.168934.r331623

**Published:** 2024-10-23

**Authors:** Sarah Roffe

**Affiliations:** 1 Agricultural Research Council, Pretoria, South Africa

This article looks really good and I have no new comments. I recommend indexing.

Is the work clearly and accurately presented and does it cite the current literature?

Yes

If applicable, is the statistical analysis and its interpretation appropriate?

Partly

Are all the source data underlying the results available to ensure full reproducibility?

Yes

Is the study design appropriate and is the work technically sound?

Yes

Are the conclusions drawn adequately supported by the results?

Partly

Are sufficient details of methods and analysis provided to allow replication by others?

Yes

Reviewer Expertise:

I am a climatologist. Within this field, and relevant to this study, I focus on outdoor thermal comfort (stress) as one aspect of my research.

I confirm that I have read this submission and believe that I have an appropriate level of expertise to confirm that it is of an acceptable scientific standard.

# Reviewer report: Reviewer response for version 3

**DOI:** 10.5256/f1000research.168934.r331621

**Published:** 2024-10-22

**Authors:** Brian Vant-Hull

**Affiliations:** 1 The City College of New York, New York, New York, USA

I feel the submission has been greatly improved, and nothing sets off alarms as I read it.  There's also a number of nice details that have been added making it an enjoyable read.

 Whether the authors mention the statistical software is up to them, I merely point out that a standard deviation is the same whether calculated by R or Python, so there is no need for the extra information.

Is the work clearly and accurately presented and does it cite the current literature?

Yes

If applicable, is the statistical analysis and its interpretation appropriate?

Yes

Are all the source data underlying the results available to ensure full reproducibility?

Yes

Is the study design appropriate and is the work technically sound?

Partly

Are the conclusions drawn adequately supported by the results?

Partly

Are sufficient details of methods and analysis provided to allow replication by others?

Yes

Reviewer Expertise:

Urban heat island, Indoor environments

I confirm that I have read this submission and believe that I have an appropriate level of expertise to confirm that it is of an acceptable scientific standard.

# Reviewer report: Reviewer response for version 2

**DOI:** 10.5256/f1000research.162963.r286765

**Published:** 2024-06-12

**Authors:** Brian Vant-Hull

**Affiliations:** 1 The City College of New York, New York, New York, USA

Overall: This could use a few additions to data analysis as well as some changes to the text as noted below.  Acceptable after some revisions.

 Abstract

 - refer to indoor temperatures relative to outdoor.  Right now the abstract only compares insulated to non insulated.

 Introduction

 The statement that metal "absorbs heat" is extremely unphysical and not even present in the cited reference.  A more physically correct statement is that metal conducts heat very efficiently, so when the surface absorbs sunlight the surface heat is rapidly conducted to the interior. This is why not only insulation but light exterior paint is required to reduce interior temperatures.  I'm disappointed that paint is not even mentioned throughout the article as it affects the main mode of energy transfer affecting interior temperature.  From the diagram it appears that all containers had the same paint, so though it wasn't a factor in this study, the fact that they all had the same external properties should at least be mentioned.

 Methods

 - though I approve of the use of apparent temperature for evaluating human comfort, it should be noted that windspeed is highly variable in urban environments due to wind shadowing, so should not be taken too seriously.  For this reason a comparison of indoor versus outdoor temperatures should also be included (this was referred to in the discussion but not shown in the results).  Since the water vapor pressure changes very little between indoor and outdoors it's actually not a vital part of the comparison between indoor and outdoor environments.

 - There's no reason to mention that statistical analysis was done using R.  Math is math.  It's like stating the brand of hammer used to build a house.  Irrelevant.

 Results

 - Figure 3: if you measured 10 containers, why are only 7 included here?  It's stated that this is a random selection, but if you can do 7 you should definitely do 10.  This suggests that there's a reason 3 were eliminated, and it wasn't random at all.  The results make clear that this was an ibutton issue. Just state this fact in the caption.

 - Figure 4: it should be stated that these are the average interior apparent temperatures, with the average applied both over all containers and over all days. 

 - The statement that "indoor AT showed greater variability compared to outdoor AT with steep increases observed in the morning and sharp declines in the afternoon" is wrong.  It's outdoor AT that shows the greater variability. This is typical for indoor temperatures due to the heat capacity of structures. Since your conclusion got this right this must be simple one word error.

 - It should be pointed out in figure 5 that the daily average AT for insulated versus non insulated is nearly the same; the benefit of insulation is it reduces the peaks.  I think points showing the daily averages of the two data sets would be a nice addition to this graph.

 - A large effect on indoor temperatures is sun exposure.  This will vary depending on urban geometry, but higher floors typically have more sun exposure and so are hotter.  Is this a trend you can report on with your data set?

 Discussion

 - the 7 degree difference in temperature between indoor and outdoor appears here but not in the results.  There should be a separate graph of indoor versus outdoor T (not just AT).

 - I suspect the Badung results used dark painted containers, possibly with less window area.  The link doesn't work so I haven't checked this.

 - The third and fith paragraphs do not refer to the current work so probably belongs to the introduction rather than discussion.

 Conclusion

 - This section should be where readers who are pressed for time can jump to for a quick summary of your actual numerical and qualitative results, NOT a set of platitudes.  WHAT DID YOU DISCOVER?

 References

 I know reference 19 link doesn't work, the others should be checked as well.

Is the work clearly and accurately presented and does it cite the current literature?

Yes

If applicable, is the statistical analysis and its interpretation appropriate?

Yes

Are all the source data underlying the results available to ensure full reproducibility?

Yes

Is the study design appropriate and is the work technically sound?

Partly

Are the conclusions drawn adequately supported by the results?

Partly

Are sufficient details of methods and analysis provided to allow replication by others?

Yes

Reviewer Expertise:

Urban heat island, Indoor environments

I confirm that I have read this submission and believe that I have an appropriate level of expertise to confirm that it is of an acceptable scientific standard, however I have significant reservations, as outlined above.

# Response

**Published:** 2024-07-08

**Authors:** Caradee Wright

**Affiliations:** South African Medical Research Council, South Africa

Response

 Overall: This could use a few additions to data analysis as well as some changes to the text as noted below.  Acceptable after some revisions.

 Abstract

 - refer to indoor temperatures relative to outdoor.  Right now the abstract only compares insulated to non insulated.

 Thank you, this suggestion has now been incorporated in the abstract – “Indoor AT was found to be 7°C higher on average when compared to outdoor AT.”

 Introduction

 The statement that metal "absorbs heat" is extremely unphysical and not even present in the cited reference.  A more physically correct statement is that metal conducts heat very efficiently, so when the surface absorbs sunlight the surface heat is rapidly conducted to the interior. This is why not only insulation but light exterior paint is required to reduce interior temperatures.  I'm disappointed that paint is not even mentioned throughout the article as it affects the main mode of energy transfer affecting interior temperature.  From the diagram it appears that all containers had the same paint, so though it wasn't a factor in this study, the fact that they all had the same external properties should at least be mentioned.

**Response: **This is a valid comment, it has been instituted in the introduction as suggested – “Although Corten steel can withstand different weather elements, it produces high thermal conductivity which enables it to absorb heat more efficiently transmitting it from the exterior of the building to the interior, which can make the interior extremely hot and uncomfortable for humans.”

 Yes, all containers had the same external dark paint color. In the method section of the study where the building is described, the paint details have been incorporated as per the reviewer's suggestion - All shipping containers were painted in dark colours that are either dark blue or dark green.  Paint plays a factor in the heating and cooling of container buildings and to incorporate this important highlight in the article, this has been included in the discussion part of the article as a suggestion – “Window shading with awnings or blinds may also help maintain cool indoor temperatures and the application of light-colored exterior paint can reduce interior temperatures.”

 Methods

 - though I approve of the use of apparent temperature for evaluating human comfort, it should be noted that windspeed is highly variable in urban environments due to wind shadowing, so should not be taken too seriously.  For this reason a comparison of indoor versus outdoor temperatures should also be included (this was referred to in the discussion but not shown in the results).  Since the water vapor pressure changes very little between indoor and outdoors it's actually not a vital part of the comparison between indoor and outdoor environments.

**Response:** A comparison of indoor and outdoor AT has been conducted and mentioned in the results section and under the “Apparent temperature findings section.” Figure 4 also shows trends in hourly indoor (blue line) and outdoor (red line) during the study period from 12 September to 25 September 2021.

 - There's no reason to mention that statistical analysis was done using R.  Math is math.  It's like stating the brand of hammer used to build a house.  Irrelevant.

**Response:** Mentioning the statistical software in the methods section of this study is important as it allows another researcher to replicate our study, and it helps the reader better understand the study results.

 Results

 - Figure 3: if you measured 10 containers, why are only 7 included here?  It's stated that this is a random selection, but if you can do 7 you should definitely do 10.  This suggests that there's a reason 3 were eliminated, and it wasn't random at all.  The results make clear that this was an ibutton issue. Just state this fact in the caption.

**Response:** The word random has been removed from the caption, thank you for highlighting this.

 - Figure 4: it should be stated that these are the average interior apparent temperatures, with the average applied both over all containers and over all days. 

**Response: **The caption for Figure 4 has been amended – “Average trends in hourly interior apparent temperatures  (blue line) with the average applied both overall containers and over all days and outdoor (red line) during the study period from 12 September to 25 September 2021.

 - The statement that "indoor AT showed greater variability compared to outdoor AT with steep increases observed in the morning and sharp declines in the afternoon" is wrong.  It's outdoor AT that shows the greater variability. This is typical for indoor temperatures due to the heat capacity of structures. Since your conclusion got this right this must be simple one word error.

**Response:** Thank you for pointing out this error, this sentence has now been amended – “Also, outdoor AT showed greater variability compared to fluctuated more than indoor AT with steep increases observed in the morning and sharp declines steep decreases recorded in the afternoon.”

 - It should be pointed out in figure 5 that the daily average AT for insulated versus non insulated is nearly the same; the benefit of insulation is it reduces the peaks.  I think points showing the daily averages of the two data sets would be a nice addition to this graph.

**Response: **Thank you for your comment. We have amended the text but we have not changed the plot.

 - A large effect on indoor temperatures is sun exposure.  This will vary depending on urban geometry, but higher floors typically have more sun exposure and so are hotter.  Is this a trend you can report on with your data set?

**Response:** Unfortunately, it is not a trend we can report on here.

 Discussion

 - the 7 degree difference in temperature between indoor and outdoor appears here but not in the results.  There should be a separate graph of indoor versus outdoor T (not just AT).

**Response:** We thank the reviewer for their comment but we would prefer to not confuse the reader by adding an additional temperature-only plot.

 - I suspect the Badung results used dark painted containers, possibly with less window area.  The link doesn't work so I haven't checked this.

**Response:** The link to this study has been corrected: Doi: 10.2991/assehr.k.201009.001

 There is no mention of the fewer window area and dark painted containers in the article only the use of air conditioning

 - The third and fith paragraphs do not refer to the current work so probably belongs to the introduction rather than discussion.

**Response: **The third paragraph was moved to the introduction, however, the fifth paragraph links to the findings of this study

 Conclusion

 - This section should be where readers who are pressed for time can jump to for a quick summary of your actual numerical and qualitative results, NOT a set of platitudes.  WHAT DID YOU DISCOVER?

**Response:** The conclusion has been amended to incorporate the study findings – “Based on the results obtained from this study, it can be concluded that occupants of Drivelines studios are at risk of experiencing adverse heat-related symptoms as the apparent temperature calculations exceeded the four symptoms band of caution, extreme caution, danger and extreme danger. These symptoms are associated with fatigue, heat cramps, heat exhaustion and the possibility of a heat stroke. If the container-based buildings are adequately insulated, then temperatures inside these buildings can be controlled.”

 References

 I know reference 19 link doesn't work, the others should be checked as well.

**Response:** The reference link for (19) has been corrected.

# Reviewer report: Reviewer response for version 2

**DOI:** 10.5256/f1000research.162963.r257740

**Published:** 2024-03-29

**Authors:** Lee Yee Yong

**Affiliations:** 1 Department of Civil Engineering, Faculty of Engineering, Universiti Malaysia Sarawak, Kota Samarahan, Sarawak, Malaysia

Good improvemant from the authors and they did address well all the comments. I would suggest to approve this version of the manuscript.

Is the work clearly and accurately presented and does it cite the current literature?

Yes

If applicable, is the statistical analysis and its interpretation appropriate?

Partly

Are all the source data underlying the results available to ensure full reproducibility?

Partly

Is the study design appropriate and is the work technically sound?

Yes

Are the conclusions drawn adequately supported by the results?

Yes

Are sufficient details of methods and analysis provided to allow replication by others?

Yes

Reviewer Expertise:

Thermal behaviour in building material, thermal comfort study.

I confirm that I have read this submission and believe that I have an appropriate level of expertise to confirm that it is of an acceptable scientific standard.

# Reviewer report: Reviewer response for version 1

**DOI:** 10.5256/f1000research.152205.r206272

**Published:** 2023-10-06

**Authors:** Sarah Roffe

**Affiliations:** 1 Agricultural Research Council, Pretoria, South Africa

This article, assessing indoor (and outdoor) thermal comfort in container buildings, is very relevant in the context of South Africa's housing crisis and the need to explore alternative, cheaper housing options while bearing in mind thermal health-related risks associated with housing options. Academically this article provides a good contribution to the limited literature regarding thermal comfort (stress) in a South African context, and it is important to inform South African developers (housing and non-housing developers). Thus, there is merit in its publication. However, before publication I have minor (perhaps even major in some instances) suggestions that I believe can significantly improve the article content and presentation:

1. Check the author affiliations - I think no 1 & 2 should be the same.
2. Abstract, background - The title refers to container buildings for housing and business yet the background is more focused on housing. Perhaps you could adjust the wording to 'Buildings (for housing and business purposes) that can protect humans...'
3. Abstract, method - To contextualise the results and their repetitiveness, add in the sampling period duration.
4. Abstract, results - When noting the AT range it will be valuable to provide the AT classification as well so that a reader can contextualise the classified body effect.
5.  Abstract, results - In your sample, there was only one non-insulated unit. Compared to the insulated units, there was certainly a difference, but care must be taken to highlight this caveat.
6. Abstract, results - n = ? for the feeling tired or weak statistic.
7. Abstract, conclusion - Although Figure 4 shows that on average insulated units have lower variation in AT (compared to the one non-insulated unit), Table 2 results suggest that the recommendation of insulated units should be assessed further (compare results for units 5, 9 [NI] and 10). This certainly suggests that above insulation, there may be other factors influencing the AT values, thus I would rather note that tentatively, the results suggest that units should be insulated. And in the conclusion I would make a note that further research on this topic is very much needed. You could also note that air conditioning is needed - of course as you highlighted, solar air conditioning would be most viable given the current electricity crisis in South Africa.
8. Abstract, conclusion - Perhaps this is an addition for the conclusion in the article... The value of your study in educating those building these container buildings should also be highlighted as it is largely their building specifications that will determine whether the building will be suitable in light of thermal health related risks.
9. Introduction - This section only focuses on the consideration of housing, yet many people spend a large proportion of their time in a business context in buildings. Provide some consideration thereof in the introduction.
10. Introduction - First word improved is perhaps misleading. Improved compared to? Perhaps use good as an alternative word.
11. Introduction - Add bracket after '...(the remaining 5% remains unknown
12. Introduction - First sentence of second paragraph needs a reference. Consider checking whether references are included where needed throughout the manuscript.
13. Methods, Study design - Provide further detail of the STROBE reporting guideline for a reader who does not know about it.
14. Methods, Study design - Consider adding in a study site map to show the location of the container units, especially in relation to the SAWS weather station used.
15. Methods, Study design - Why was the period of 12-25 Sep 2021 used for sampling? I would provide justification in text.
16. Methods, Temperature measurements - The heading of this section is misleading as you did not only record temperatures. Instead title the section Meteorological measurements?
17. Methods, Temperature measurements - More information is required for the iButtons loggers. How often do they provide measurements?
18. Methods, Temperature measurements - More information is required for the SAWS weather station. Which weather station was this? How far is it from the container building? etc. This is why I suggested a study site map - perhaps consider adding landuse/cover and elevation to give the reader context on how representative this station is.
19. Methods, Apparent temperature calculations and heat-health risks - Why have you considered the apparent temperature method? Provide some justification, especially because the metric used to assess thermal comfort can strongly influence results (see: Simpson, C.H., Brousse, O., Ebi, K.L. and Heaviside, C., 2023. Commonly used indices disagree about the effect of moisture on heat stress. *npj Climate and Atmospheric Science*, *6*(1), p.78.). I am not suggesting to change the method, but I am suggesting you acknowledge this consideration of metrics and their potential influence on results, especially in your final discussion paragraph.
20. Methods, Survey questionnaire - All participants were considered exposed. Exposed to what? Be more specific.
21. Methods, Statistical analysis - Provide detail on how much data were missing. A supplementary table should suffice.
22. Methods, Statistical analysis - You report on using the paired t-test, but in the results you do not show any results thereof. Either add these results or omit noting that you used the paired t-test.
23. Results - The first paragraph provides information more suited to the methods. I would incorporate this information in the methods.
24. Results, Apparent temperature findings - Avoid referral to ambient temperature in the context of your work. Rather refer to indoor or outdoor temperature as ambient temperature refers to the temperature of the immediate surroundings which can be indoor or outdoor.
25. Results, Apparent temperature findings - Over what period (i.e. years) and at what resolution (i.e. daily or monthly) are the September temperatures you refer to, and is this from the SAWS station you used? Take care not to omit such important information.
26. Results, Apparent temperature findings - 'Indoor AT measurements made in the container units displayed similar diurnal patterns with the warmest temperatures occurring in the mid-afternoon ( Figure 2).' Your figure does not show this?
27. Results, Apparent temperature findings - Your figure captions are slightly incorrect. You cannot refer to a trend with only 14 days of data. A trend in a climatological context is a measurement of 'change' over at least a 30 year period. You are only showing average diurnal variation in AT for figure 2-4.
28. Results, Apparent temperature findings - Provide more reference to the figures in your results as it not always clear as to which figure you are referring to.
29. Results, Apparent temperature findings - At the end of the second paragraph, you say that indoor AT fluctuated more than outdoor AT. This is not true according to Figure 3.
30. Results, Apparent temperature findings - Figure 2 could be be improved on. I would ideally indicate which units were insulated and which unit was not insulated, and for better comparability of AT, it is necessary to use the same y-axis range. Also, consider providing a straight lines from the y-axis to indicate the different AT thresholds for Table 1. This will provide more context to your results. This last suggestion also applies for figure 3.
31. Results, Apparent temperature findings - You note that AT for insulated vs non-insulated differed by ~2-9 degrees C. Were the values statistically different (i.e. what are the t-test results)?
32. Results, Apparent temperature findings - You could add an indication of whether the unit was insulated or not for table 2 to help interpret the results of this table. Also, as previously mentioned, it does not provide compelling evidence that insulated units are better, especially since your sample size is small with only one unit that is not insulated.
33. Results, Questionnaire findings - The second sentence should be reworded.
34. Results overall - No t-test results are reported despite mention in the methods.
35. Results overall - consider adding more reporting on the results to strengthen this section.
36. Discussion - 'This study has shown the significant variation in indoor temperatures in shipping container units used for living and business purposes in the metropolitan city of Johannesburg.' This sentence is misleading. You have used significant in an incorrect manner. This is a statistical term and should only be used if there is statistical backing for which there is not in this paper. Consider rewording this sentence to more appropriately align with what you have truly shown in your study.
37. Discussion - 'On average, indoor temperatures were consistently warmer than outdoor temperatures...' How much warmer? A sentence like this should always be backed up by numerical estimates.
38. Discussion - 'All units in which measurements were made had exceedances of the heat-health risk threshold values related to ‘caution’, ‘extreme caution’, ‘danger’ and ‘extreme danger’.' According to Table 2 and Figure 2, this sentence is misleading and perhaps even incorrect. If I am interpreting the results correctly, all units recorded caution levels, but only 3 recorded levels above this?
39. Discussion - 'Two container units had the highest frequency of occurrences of temperatures reaching the ‘caution’ (27–32°C) threshold – one unit was insulated and the other was not – that are associated with fatigue possible with prolonged exposure and/or physical activity'. This sentence also seems to be misleading and perhaps incorrect.
40. Discussion - 'This suggests that insulation might not be the sole solution for maintaining consistent temperatures in shipping container units.' But you suggest insulation in the abstract? Some of your statements are contradictory.
41. Discussion - When referring to results in the discussion, it is good practice to refer to the Table or Figure showing the results.
42. Discussion - Third paragraph first sentence needs a reference. In fact, I recommend more actively referencing in your work.
43. Discussion - 'South Africa is projected to experience an increase in average ambient temperature between 4–6°C by 2100. ^17^ ' This should be reference 18.
44. Discussion - 'Furthermore, a heat stress assessment study found that the CBD of the city of Johannesburg, which is where the containers were located, exhibited urban heat island characteristics due to high building densities and sparse vegetation. ' This sentence needs a reference.
45. Discussion - You suggest two iButtons in case one fails. This could also be useful for comparability to determine accuracy of the readings.
46. Conclusion - You study opens doors for much more research to be undertaken. I think it is important that you highlight this so that a reader can better contextualise your results and also so they can consider how representative your results are. Important for this is assessing seasonality and also taking readings over multiple years to account for variability.

Is the work clearly and accurately presented and does it cite the current literature?

Yes

If applicable, is the statistical analysis and its interpretation appropriate?

Partly

Are all the source data underlying the results available to ensure full reproducibility?

Yes

Is the study design appropriate and is the work technically sound?

Yes

Are the conclusions drawn adequately supported by the results?

Partly

Are sufficient details of methods and analysis provided to allow replication by others?

Yes

Reviewer Expertise:

I am a climatologist. Within this field, and relevant to this study, I focus on outdoor thermal comfort (stress) as one aspect of my research.

I confirm that I have read this submission and believe that I have an appropriate level of expertise to confirm that it is of an acceptable scientific standard, however I have significant reservations, as outlined above.

## References

[1] : Commonly used indices disagree about the effect of moisture on heat stress. *npj Climate and Atmospheric Science* .2023;6(1) : 10.1038/s41612-023-00408-0 10.1038/s41612-023-00408-0 PMC761550438204467

# Response

**Published:** 2024-02-20

**Authors:** Caradee Wright

**Affiliations:** South African Medical Research Council, South Africa

**Reviewer 2**

 APPROVED WITH RESERVATIONS

 This article, assessing indoor (and outdoor) thermal comfort in container buildings, is very relevant in the context of South Africa's housing crisis and the need to explore alternative, cheaper housing options while bearing in mind thermal health-related risks associated with housing options. Academically this article provides a good contribution to the limited literature regarding thermal comfort (stress) in a South African context, and it is important to inform South African developers (housing and non-housing developers). Thus, there is merit in its publication. However, before publication I have minor (perhaps even major in some instances) suggestions that I believe can significantly improve the article content and presentation:

  

1. Check the author affiliations - I think no 1 & 2 should be the same.

**Response:** The author’s affiliations are accurately reported and comprise of two different government departments that the author has affiliations with.

  

1. Abstract, background - The title refers to container buildings for housing and business yet the background is more focused on housing. Perhaps you could adjust the wording to 'Buildings (for housing and business purposes) that can protect humans...'

**Response:**  We have adjusted the introduction to better reflect the focus on both housing and business aspects of container buildings as follows:

 “Properly designed and constructed  residential, business and public service spaces can help prevent disease, reduce poverty, and increase quality of life. ^1,2^ Adequate, safe housing and working environments are essential in the current global context of urbanisation, ageing populations, and climate change. ^1^ In South Africa, around 80% of the population of around ~60 million people live in formal dwellings, 11% live in informal dwellings such as shacks, 4% live in traditional dwellings and the other 5% is unknown. ^3^ Government-provided, formal low-cost housing has failed to address the growing informal housing problem in South Africa. ^4^ Recently, initiatives by developers have increased the use of shipping containers to provide for South Africans who earn very little. As low-cost housing solutions, several shipping containers have been combined into a single storey or stacked to make a multi-storey block of apartments. To serve poorer communities, shipping containers have been used as clinics as well as “spaza shops” to sell essential items in townships. ^5,6 ^”

  

1. Abstract, method - To contextualise the results and their repetitiveness, add in the sampling period duration.

**Response:**  We have added the sampling period to the abstract:

 “…comprising a seven-storey block of apartments and businesses in Johannesburg, South Africa for 14 days.”

  

1. Abstract, results - When noting the AT range it will be valuable to provide the AT classification as well so that a reader can contextualise the classified body effect.

**Response:** We thank the reviewer for their comment. The definition of AT is already present in the abstract in a manner concise enough to adhere to the abstract word count as follows:

 “Median indoor apparent temperature (AT) (a combination of temperature and relative humidity)”

  

1.  Abstract, results - In your sample, there was only one non-insulated unit. Compared to the insulated units, there was certainly a difference, but care must be taken to highlight this caveat.

**Response:** We changed the wording to reflect that there was one uninsulated unit as follows: “than the uninsulated unit”

  

1. Abstract, results - n = ? for the feeling tired or weak statistic.

**Response:** The “n= 62” has been removed from “experiencing headaches,” thus ensuring uniformity in the reporting of results in the abstract.

  

1. Abstract, conclusion - Although Figure 4 shows that on average insulated units have lower variation in AT (compared to the one non-insulated unit), Table 2 results suggest that the recommendation of insulated units should be assessed further (compare results for units 5, 9 and 10). This certainly suggests that above insulation, there may be other factors influencing the AT values, thus I would rather note that tentatively, the results suggest that units should be insulated. And in the conclusion I would make a note that further research on this topic is very much needed. You could also note that air conditioning is needed - of course as you highlighted, solar air conditioning would be most viable given the current electricity crisis in South Africa.

**Response:** The conclusion within the abstract has been edited as follows:

 “Residents, tenants, or business owners using shipping containers should consider insulation installation and adequate windows/air conditioning for ventilation, especially in hot climates. Further research and awareness regarding heat-health risks of living or working in these spaces is needed.”

  

1. Abstract, conclusion - Perhaps this is an addition for the conclusion **in the article**... The value of your study in educating those building these container buildings should also be highlighted as it is largely their building specifications that will determine whether the building will be suitable in light of thermal health related risks.

**Response:** We thank the reviewer for their comment. We have addressed this concern in the conclusion as follows:

 “The findings of future studies that analyse optimisation of thermal comfort in shipping containers should be used to advocate for and educate people who oversee the adaptation of shipping containers for either residential or business uses.”

  

1. Introduction - This section only focuses on the consideration of housing, yet many people spend a large proportion of their time in a business context in buildings. Provide some consideration thereof in the introduction.

**Response:** The introduction has been edited to reflect this concern as follows:

 “Properly designed and constructed  residential,  business and public service spaces can help prevent disease, reduce poverty, and increase quality of life. ^1,2^ Adequate, safe housing and working environments are essential in the current global context of urbanisation, ageing populations, and climate change. ^1^ In South Africa, around 80% of the population of around ~60 million people live in formal dwellings, 11% live in informal dwellings such as shacks, 4% live in traditional dwellings and the other 5% is unknown. ^3^ Government-provided, formal low-cost housing has failed to address the growing informal housing problem in South Africa. ^4^ Recently, initiatives by developers have increased the use of shipping containers to provide for South Africans who earn very little. As low-cost housing solutions, several shipping containers have been combined into a single storey or stacked to make a multi-storey block of apartments. To serve poorer communities, shipping containers have been used as clinics as well as “spaza shops” to sell essential items in townships. ^5,^ ^6^.”

  

1. Introduction - First word improved is perhaps misleading. Improved compared to? Perhaps use good as an alternative word.

**Response: **The wording of the first sentence of the introduction has been changed as follows:

 “Improved housing conditions” was changed to “Properly designed and constructed residential,  business and public service spaces”

  

1. Introduction - Add bracket after '.(the remaining 5% remains unknown

**Response: **We thank for reviewer for spotting this error. This error has been corrected in the document.

  

1. Introduction - First sentence of second paragraph needs a reference. Consider checking whether references are included where needed throughout the manuscript.

**Response:** We thank the reviewer for their insight. We have added a suitable reference to the sentence.

  

1. Methods, Study design - Provide further detail of the STROBE reporting guideline for a reader who does not know about it.

**Response:** Further clarity has been added on the value of the using STROBE guidelines to enhance the quality of observational study data:

 “Strengthening the Reporting of Observational studies in Epidemiology (STROBE) guidelines were used to plan and execute this study. The STROBE guidelines ensure the highest quality of observational study data and consists of 22 checklist items that should be addressed in a study. ^16^”

  

1. Methods, Study design - Consider adding in a study site map to show the location of the container units, especially in relation to the SAWS weather station used.

**Response:** Thank you for your insightful feedback and suggestion to include a study site map showcasing the container units' location in relation to the SAWS weather station.

 We have included a study site map and it is now Figure 1 in the revised manuscript.

  

1. Methods, Study design - Why was the period of 12-25 Sep 2021 used for sampling? I would provide justification in text.

**Response:** A sentence has been added to justify the sampling period:

 “The sampling took place in September because it is spring time in South Africa, and high temperatures were recorded in previous years during this month.”

  

1. Methods, Temperature measurements - The heading of this section is misleading as you did not only record temperatures. Instead title the section Meteorological measurements?

**Response:** The heading has been changed to “Meteorological measurements”

  

1. Methods, Temperature measurements - More information is required for the iButtons loggers. How often do they provide measurements?

**Response: **The frequency of measurements from the iButtons has been added to the description:

 “iButtons are small, portable sensors that record hourly temperature and relative humidity”

  

1. Methods, Temperature measurements - More information is required for the SAWS weather station. Which weather station was this? How far is it from the container building? etc. This is why I suggested a study site map - perhaps consider adding landuse/cover and elevation to give the reader context on how representative this station is.

**Response:** We thank the reviewer for this comment. The location of the weather station is now given in Figure 1 of the revised manuscript.

  

1. Methods, Apparent temperature calculations and heat-health risks - Why have you considered the apparent temperature method? Provide some justification, especially because the metric used to assess thermal comfort can strongly influence results (see: Simpson, C.H., Brousse, O., Ebi, K.L. and Heaviside, C., 2023. Commonly used indices disagree about the effect of moisture on heat stress. *npj Climate and Atmospheric Science*, *6*(1), p.78.). I am not suggesting to change the method, but I am suggesting you acknowledge this consideration of metrics and their potential influence on results, especially in your final discussion paragraph.

**Response:** We would like to clarify that the paper by Simpson et al. (2023) raises important considerations about the potential influence of radiation and wind on the calculation of real feel temperature using different metrics on heat stress assessment. The paper emphasizes that when calculating indoor ambient temperature, wind and radiation do not need to be considered. Thus, we used a suitable equation to calculate indoor and outdoor real feel temperature.

 Nevertheless, we acknowledge the significance of addressing the potential impact of the chosen metric on our results.

 Here are some quotes from other articles that used AT to calculate real feel temperatures in indoor environments:

*First citation: *Naicker, N., Teare, J., Balakrishna, Y., Wright, C. Y., & Mathee, A. (2017). Indoor Temperatures in Low Cost Housing in Johannesburg, South Africa. *International Journal of Environmental Research and Public Health*, *14*(11). https://doi.org/10.3390/ijerph14111410

 “Apparent temperature is an indicator of thermal sensation, can be used in indoor settings [28] and has been used before when considering the relationship between heat and thermal comfort [29,30].”

 28. Nguyen, J.L.; Schwartz, J.; Dockery, D.W. The relationship between indoor and outdoor temperature, relative humidity, and absolute humidity. Indoor Air 2014, 24, 103–112.

 29. Baccini, M.; Biggeri, A.; Accetta, G.; Kosatsky, T.; Katsouyanni, K.; Analitis, A.; Anderson, H.R.; Bisanti, L.; D’Ippoliti, D.; Danova, J.; et al. Heat effects on mortality in 15 European cities. Epidemiology 2008, 19, 711–719.

 30. Bell, M.L.; O’Neill, M.S.; Ranjit, N.; Borja-Aburto, V.H.; Cifuentes, L.A.; Gouveia, N.C. Vulnerability to heat-related mortality in Latin America: A case-crossover study in Sao Paulo, Brazil, Santiago, Chile and Mexico City, Mexico. Int. J. Epidemiol. 2008, 37, 796–804.

*Second citation*: Kapwata, T., Gebreslasie, M. T., Mathee, A., & Wright, C. Y. (2018). Current and Potential Future Seasonal Trends of Indoor Dwelling Temperature and  Likely Health Risks in Rural Southern Africa. *International Journal of Environmental Research and Public Health*, *15*(5). https://doi.org/10.3390/ijerph15050952

 “AT is an adjustment to the ambient temperature based on the level of relative humidity and it is

 deemed a measure of how humans actually perceive or feel temperature. AT is the most frequently used indicator of probable human physical reaction to weather conditions and several studies have used AT to examine the association between health and high temperature [46–49].”

 46. Garland, R.M.; Matooane, M.; Engelbrecht, F.A.; Bopape, M.J.M.; Landman, W.A.; Naidoo, M.; Merwe, J.V.D.; Wright, C.Y. Regional projections of extreme apparent temperature days in Africa and the related potential risk to human health. Int. J. Environ. Res. Public Health 2015, 12, 12577–12604.

 47. Harlan, S.L.; Brazel, A.J.; Prashad, L.; Stefanov, W.L.; Larsen, L. Neighborhood microclimates and vulnerability to heat stress. Soc. Sci. Med. 2006, 63, 2847–2863.

 48. Watts, J.D.; Kalkstein, L.S. The development of a warm-weather relative stress index for environmental applications. J. Appl. Meteorol. 2004, 43, 503–513. [CrossRef]

 49. Baccini, M.; Biggeri, A.; Accetta, G.; Kosatsky, T.; Katsouyanni, K.; Analitis, A.; Anderson, H.R.; Bisanti, L.; D’ippoliti, D.; Danova, J.; et al. Heat effects on mortality in 15 European cities. Epidemiology 2008, 19, 711–719.

  

1. Methods, Survey questionnaire - All participants were considered exposed. Exposed to what? Be more specific.

**Response:** We apologize for the ambiguity in the statement and have removed the sentence.

  

1. Methods, Statistical analysis - Provide detail on how much data were missing. A supplementary table should suffice.

**Response: **We thank the reviewer for addressing this concern. By acknowledging and appropriately handling missing data, we aim to uphold the integrity of our study and contribute to the broader scientific discourse with accurate and reliable results.

 To address the issue of missing data we have mentioned that 50% of the data were missing.

  

1. Methods, Statistical analysis - You report on using the paired t-test, but in the results you do not show any results thereof. Either add these results or omit noting that you used the paired t-test.

**Response: **We thank the reviewer for this comment and after deliberations, we realised that the t-test was not performed due to small sample size and was not removed from the original draft of the manuscript. We apologize for this oversight, and we have now removed the mention of a t-test from the methods.

  

1. Results - The first paragraph provides information more suited to the methods. I would incorporate this information in the methods.

**Response: **This paragraph was rephrased and added to the methods section under the subheading “Survey questionnaire”:

 “We administered a short survey questionnaire to occupants of the units after obtaining their informed consent.”

 “A total of 62 participants (58%) answered the survey. Other residents and business owners (n=45) in the building were not available to participate during the study period.”

  

1. Results, Apparent temperature findings - Avoid referral to ambient temperature in the context of your work. Rather refer to indoor or outdoor temperature as ambient temperature refers to the temperature of the immediate surroundings which can be indoor or outdoor.

**Response:** We apologize for the ambiguity in the use of the word “ambient” and we have corrected this throughout the manuscript.

  

1. Results, Apparent temperature findings - Over what period (i.e. years) and at what resolution (i.e. daily or monthly) are the September temperatures you refer to, and is this from the SAWS station you used? Take care not to omit such important information.

**Response:** We thank the reviewer for this query and we would like to clarify that the SAWS data was within the same time frame as the iButtons measurements comparing the outdoor and indoor temperature during the month of September. Thus, the information was not omitted, and the period of the study was given in the methods section. We have clarified this within the manuscript in the methods section as follows under “Meteorological measurements”:

 “Meteorological data within the study period (12 September to 25 September 2021) were provided at 10-minute intervals which were converted to hourly interval calculations.”

  

1. Results, Apparent temperature findings - Indoor AT measurements made in the container units displayed similar diurnal patterns with the warmest temperatures occurring in the mid-afternoon ( Figure 2). Your figure does not show this?

**Response: **Upon careful re-evaluation of Figure 2, the figure illustrates the diurnal patterns of indoor apparent temperature measurements, with the warmest temperatures observed in the mid-afternoon.

  

1. Results, Apparent temperature findings - Your figure captions are slightly incorrect. You cannot refer to a trend with only 14 days of data. A trend in a climatological context is a measurement of 'change' over at least a 30 year period. You are only showing average diurnal variation in AT for figure 2-4.

**Response:** To address your concern and ensure clarity, we modified the captions to use the term "changes," which better aligns with the temporal scope of our study.

  

1. Results, Apparent temperature findings - Provide more reference to the figures in your results as it not always clear as to which figure you are referring to.

Response: We have carefully considered your suggestion and upon re-evaluation of the results section we have noted the following:

- We have explicit references to the relevant figures in the text.
- We have detailed figure citations, ensuring that readers can easily identify and cross-reference the information presented in the text with the corresponding figures.
- We have figure captions to provide clear descriptions of the key findings presented in each figure.

We believe that these elements significantly provide an alignment between the narrative in the Results section and the corresponding figures, thereby facilitating a seamless understanding of our findings. We hope that these characteristics of our results section effectively address your concerns so that you are assured that we have provided an overall seamless reading experience for our audience.

  

1. Results, Apparent temperature findings - At the end of the second paragraph, you say that indoor AT fluctuated more than outdoor AT. This is not true according to Figure 3.

**Response:**  We apologise for the incorrect use of the term “fluctuated” and we have edited the statement to be more accurate as follows:

  "Indoor AT showed greater variability compared to outdoor AT, with steep increases observed in the morning and sharp declines in the afternoon."

  

1. Results, Apparent temperature findings - Figure 2 could be improved on. I would ideally indicate which units were insulated and which unit was not insulated, and for better comparability of AT, it is necessary to use the same y-axis range. Also, consider providing a straight lines from the y-axis to indicate the different AT thresholds for Table 1. This will provide more context to your results. This last suggestion also applies for figure 3.

**Response: **We have revised figure 2 so that all plots have the same y-axis, however, we prefer not to add threshold lines as the graphs will become very cluttered.


** **

1. Results, Apparent temperature findings - You note that AT for insulated vs non-insulated differed by ~2-9 degrees C. Were the values statistically different (i.e. what are the t-test results)?

**Response: **We thank the reviewer for this comment and after deliberations, we realised that the t-test was not performed due to small sample size and was not removed from the original draft of the manuscript. We apologize for this oversight, and we have now removed the mention of a t-test from the methods. Statistical significance could not be elaborated.

  

1. Results, Apparent temperature findings - You could add an indication of whether the unit was insulated or not for table 2 to help interpret the results of this table. Also, as previously mentioned, it does not provide compelling evidence that insulated units are better, especially since your sample size is small with only one unit that is not insulated.

**Response:** An indication of which units were insulated and which were uninsulated were added as a footnote to Table 2.

 Regarding the comment on whether insulation was better or not, this study did not aim to elucidate the affect of insulation on shipping containers and the uninsulated container was not a control that was measured against the insulated shipping containers. We may have confused the reviewer by adding a paragraph addressing insulation of shipping containers in the introduction. Therefore, we have moved this paragraph addressing the effects on insulation on thermal comfort to the discussion.

  

1. Results, Questionnaire findings - The second sentence should be reworded.

**Response:** We thank the reviewer for this suggestion. The sentence has been edited as follows:

 “At the time of the survey, the shipping container structure was 5 years old, having been assembled in 2017, when units were available to rent or purchase. All participants of this study were utilizing the containers for less than 6 months. All surveyed participants reported that their units had windows and were occupied by one or more people.”

  

1. Results overall - No t-test results are reported despite mention in the methods.

**Response:** We thank the reviewer for this comment and after deliberations, we realised that the t-test was not performed due to small sample size and was not removed from the original draft of the manuscript. We apologize for this oversight, and we have now removed the mention of a t-test from the methods.

  

1. Results overall - consider adding more reporting on the results to strengthen this section.

**Response:** We sincerely appreciate the thoughtful feedback, and we have diligently incorporated these valuable suggestions into the results section. In response to the specific comment regarding the need for additional reporting, we have enhanced the comprehensiveness of our results presentation by meticulously addressing each and every comment and incorporating pertinent adjustments.

  

1. Discussion - 'This study has shown the significant variation in indoor temperatures in shipping container units used for living and business purposes in the metropolitan city of Johannesburg.' This sentence is misleading. You have used significant in an incorrect manner. This is a statistical term and should only be used if there is statistical backing for which there is not in this paper. Consider rewording this sentence to more appropriately align with what you have truly shown in your study.

**Response:** We apologize for the incorrect use of the word “significant” and we have rephrased the sentence as follows:

 “This study has successfully clarified the low indoor thermal comfort characteristics of shipping container units used for living and business purposes in a hot and humid environment in the metropolitan city of Johannesburg.”

  

1. Discussion - 'On average, indoor temperatures were consistently warmer than outdoor temperatures...' How much warmer? A sentence like this should always be backed up by numerical estimates.

**Response:** The numerical reference was added to the sentence:

 “Indoor temperatures were consistently warmer than outdoor temperatures (~ 7°C higher on average) and with some non-optimal high temperature exceedances presenting a reason for concern regarding people’s heat-health risks.”

  

1. Discussion - 'All units in which measurements were made had exceedances of the heat-health risk threshold values related to ‘caution’, ‘extreme caution’, ‘danger’ and ‘extreme danger’.' According to Table 2 and Figure 2, this sentence is misleading and perhaps even incorrect. If I am interpreting the results correctly, all units recorded caution levels, but only 3 recorded levels above this?

**Response:** We thank the reviewer for this comment. We have edited the sentences as follows:

 “All units in which measurements were made exceeded heat-health risk threshold values related to ‘caution’. Three units exceeded the threshold values related to ‘extreme caution’ of 33-39 ℃. One unit exceeded the threshold values related to ‘danger’ of 40-51 ℃.”

  

1. Discussion - 'Two container units had the highest frequency of occurrences of temperatures reaching the ‘caution’ (27–32°C) threshold – one unit was insulated and the other was not – that are associated with fatigue possible with prolonged exposure and/or physical activity'. This sentence also seems to be misleading and perhaps incorrect.

**Response:** We thank the reviewer for their comments and we have edited the statement as follows:

 “Two container units, one insulated and the other not had the highest frequency of temperature occurrences surpassing the ‘caution’ (27 – 32°C) threshold. These temperatures are associated with fatigue that is equivalent to prolonged heat exposure and / or physical activity.”

  

1. Discussion - 'This suggests that insulation might not be the sole solution for maintaining consistent temperatures in shipping container units.' But you suggest insulation in the abstract? Some of your statements are contradictory.

**Response: **We apologize for the confusion caused by discussing insulation of shipping containers in the introduction. We have moved this paragraph to the discussion.

  

1. Discussion - When referring to results in the discussion, it is good practice to refer to the Table or Figure showing the results.

**Response:** We thank the reviewer for their comment. After careful consideration, we would like to provide our rationale for not explicitly referring to specific tables and figures in the discussion section.

 While we understand the common practice of referencing tables and figures to enhance clarity, we have opted for a more narrative approach in our discussion to maintain a cohesive and flowing narrative. We aimed to present our findings in a manner that allows readers to follow the logical progression of our arguments without constant references to specific data visuals, that has already been done in the results section.

 However, to address your concern, we have ensured that key results are appropriately highlighted in the text, providing a clear link between our discussion points and the corresponding data in tables and figures. We believe this approach maintains readability while still allowing for a comprehensive understanding of our results.

 We hope this explanation aligns with the standards of the journal, and we are open to further suggestions or modifications if needed.

  

1. Discussion - Third paragraph first sentence needs a reference. In fact, I recommend more actively referencing in your work.

**Response:** We thank the reviewer for their comment on referencing. We acknowledge the importance of providing proper citations to support our statements and have addressed this concern by incorporating additional references throughout our work.

  

1. Discussion - 'South Africa is projected to experience an increase in average ambient temperature between 4–6°C by 2100. ^17^ ' This should be reference 18.

**Response:** We thank the reviewer for pointing out this error. We have redone all references in light of the extensive changes to the manuscript and have taken precautions to ensure that all referencing is accurate.

  

1. Discussion - 'Furthermore, a heat stress assessment study found that the CBD of the city of Johannesburg, which is where the containers were located, exhibited urban heat island characteristics due to high building densities and sparse vegetation.' This sentence needs a reference.

**Response:** We thank the reviewer for their comment. We have added the following reference: Hardy C, Nel A. Data and techniques for studying the urban heat island effect in Johannesburg. ISPRS - International Archives of the Photogrammetry, Remote Sensing and Spatial Information Sciences. XL-7/W3. 203-206. 10.5194/isprsarchives-XL-7-W3-203-2015. 2015.

  

1. Discussion - You suggest two iButtons in case one fails. This could also be useful for comparability to determine accuracy of the readings.

**Response:** We thank the reviewer for their insight. We have added this to the conclusions:

 “In future, two iButtons should be placed side-by-side to have a back-up device in the event that one logger fails to prevent data loss and to increase the accuracy and variation between the readings.”

  

1. Conclusion - You study opens doors for much more research to be undertaken. I think it is important that you highlight this so that a reader can better contextualise your results and also so they can consider how representative your results are. Important for this is assessing seasonality and also taking readings over multiple years to account for variability.

**Response:** We thank the reviewer for their insight. We have added a paragraph on future studies to the conclusions.

 “Given the projected extreme outdoor temperatures (both heat and cold) due to climate change, it is imperative to devise strategies to enhance indoor thermal comfort. This is particularly important for low- and middle-income countries, ensuring the well-being of those forced to consider cheaper building options. The information gleaned from this study can be used to conceptualise future research to solve the thermal comfort limitations of shipping containers. Characterising measures to ensure thermal comfort inside shipping container residential and business units is warranted. Solutions such as green or vegetated roofs, exterior wall insulation options, double-glazing on windows, ^21^ and solar-powered air-conditioning could be further explored and optimized. Building on insights from similar studies in different climates, researchers can delve deeper into the performance of various insulation materials, considering factors such as cost-effectiveness and sustainability. The influence of building design is especially interesting, particularly ventilation through breezes and the direction of buildings. Indoor temperatures should be a focal point for architects and urban planners aiming to create climate-resilient structures. Future research could also extend beyond temperature measurements to include physiological assessments of inhabitants for personalized exposure estimates, providing a more holistic understanding of heat-health impacts. Finally, addressing study limitations by increasing sample size, incorporating backup devices, and extending data collection periods to both summer and winter would strengthen the reliability and applicability of future research in this domain.”

  

- **Is the work clearly and accurately presented and does it cite the current literature?**

Yes

- **Is the study design appropriate and is the work technically sound?**

Yes

- **Are sufficient details of methods and analysis provided to allow replication by others?**

Yes

- **If applicable, is the statistical analysis and its interpretation appropriate?**

Partly

- **Are all the source data underlying the results available to ensure full reproducibility?**

Yes

- **Are the conclusions drawn adequately supported by the results?**

Partly

**References**

 1. Simpson C, Brousse O, Ebi K, Heaviside C: Commonly used indices disagree about the effect of moisture on heat stress. *npj Climate and Atmospheric Science*. 2023; **6** (1). Publisher Full Text

 Competing Interests

*No competing interests were disclosed.*

 Reviewer Expertise

 I am a climatologist. Within this field, and relevant to this study, I focus on outdoor thermal comfort (stress) as one aspect of my research.

**I confirm that I have read this submission and believe that I have an appropriate level of expertise to confirm that it is of an acceptable scientific standard, however I have significant reservations, as outlined above.**

# Reviewer report: Reviewer response for version 1

**DOI:** 10.5256/f1000research.152205.r206274

**Published:** 2023-10-03

**Authors:** Lee Yee Yong

**Affiliations:** 1 Department of Civil Engineering, Faculty of Engineering, Universiti Malaysia Sarawak, Kota Samarahan, Sarawak, Malaysia

This article measured the indoor temperature inside shipping containers comprising a seven-story block of apartments and businesses in Johannesburg, South Africa. This study is good as it could potentially give an important insight into another type of sustainable housing by using containers. However, the effects of temperature are essential due to its material properties on heat transfer, especially in the context of climate change.

 In the overview of the article, the authors suggest improving the introduction by highlighting the thermal behavior of the material used for the container, as it is also part of the factor contributing to indoor heat storage due to heat transfer. It is also suggested that the author include some thermal properties of the material to support your justification for the impact of heat on temperature. It is important to emphasize the needs and novelty of your contribution towards knowledge as well as promoting the usage of container buildings by overcoming the effects of temperature, especially climate change.

 In terms of methodology, the authors are advised to include the locality of the instruments within the space of the measurement. The location of the instrument is essential to validating the method of data collection. There are 10 units of containers that have been chosen. Suppose it is believed that there are 10 different surrounding environments for each of the units. The author is suggested to describe the different characteristics of the 10 units to show the temperature variation and fit to the data reporting in the results section. It will have more meaning to reflect the different environmental conditions affecting heat impacts. Besides that, this study incorporated a questionnaire survey and physical measurements. It would be nice if the author could associate the survey findings with the results from the physical measurement, where there is no discussion about it.

 Under the results section, apparent temperature findings mostly presented the data and lacked critical discussion, which reflected the significant AT difference between the units, e.g., paragraphs 3 and 4 in the results section. More references to support the judgment and the input for different environmental conditions may be helpful in this case.

Is the work clearly and accurately presented and does it cite the current literature?

Yes

If applicable, is the statistical analysis and its interpretation appropriate?

Partly

Are all the source data underlying the results available to ensure full reproducibility?

Partly

Is the study design appropriate and is the work technically sound?

Yes

Are the conclusions drawn adequately supported by the results?

Yes

Are sufficient details of methods and analysis provided to allow replication by others?

Yes

Reviewer Expertise:

Thermal behaviour in building material, thermal comfort study.

I confirm that I have read this submission and believe that I have an appropriate level of expertise to confirm that it is of an acceptable scientific standard, however I have significant reservations, as outlined above.

# Response

**Published:** 2024-02-20

**Authors:** Caradee Wright

**Affiliations:** South African Medical Research Council, South Africa

**Reviewer 1**

 1. This article measured the indoor temperature inside shipping containers comprising a seven-story block of apartments and businesses in Johannesburg, South Africa. This study is good as it could potentially give an important insight into another type of sustainable housing by using containers. However, the effects of temperature are essential due to its material properties on heat transfer, especially in the context of climate change.

**Response:** We thank the reviewer for their comments.

 2. In the overview of the article, the authors suggest improving the introduction by highlighting the thermal behavior of the material used for the container, as it is also part of the factor contributing to indoor heat storage due to heat transfer. It is also suggested that the author include some thermal properties of the material to support your justification for the impact of heat on temperature.

**Response:** We have revised the introduction to emphasize the thermal behavior of the container material and its contribution to indoor heat storage. Additionally, we have included relevant thermal properties of the material to support our justification for the impact of heat on temperature.

 “Shipping containers are made of Corten steel, which possesses the physical properties that make it weldable and rust-resistant. ^11^ Although Corten steel can withstand different weather elements, it also absorbs heat, which can make the interior extremely hot and uncomfortable for humans. ^10^”

 3. It is important to emphasize the needs and novelty of your contribution towards knowledge as well as promoting the usage of container buildings by overcoming the effects of temperature, especially climate change.

**Response: **We thank the reviewer for pointing out that we need to emphasize the importance of our study. We have made the following adjustments to the introduction:

 “The only study analysing temperatures inside shipping containers was of classrooms in Johannesburg, South Africa, which showed temperatures in excess of 40 °C during the summer. ^15^ There have been no studies to characterise thermal comfort conditions by measuring both temperature and humidity inside shipping containers converted into residences or businesses in South Africa. Therefore, we aimed to measure indoor temperature and humidity inside shipping containers comprising a seven-storey block of apartments and businesses in Johannesburg, South Africa.”

 “The study data may form a foundation for the development of guidelines and regulations required to improve the habitability of converted containers. In addition, these findings are important to inform policy making and health awareness campaigns related to living and working in shipping containers in hot climates.”

 4. In terms of methodology, the authors are advised to include the locality of the instruments within the space of the measurement. The location of the instrument is essential to validating the method of data collection. There are 10 units of containers that have been chosen. Suppose it is believed that there are 10 different surrounding environments for each of the units. The author is suggested to describe the different characteristics of the 10 units to show the temperature variation and fit to the data reporting in the results section. It will have more meaning to reflect the different environmental conditions affecting heat impacts.

**Response:** We have rephrased the information on the locality of the instruments within the container space to clarify the suggestion given by the reviewer, thereby specifying the iButton locations for accurate validation of the data collection method.

 “iButtons were uniformly installed against a cupboard, away from the walls, in all ten containers from September 12 to September 25, 2021.”

 The characteristics of the 10 selected units have been described in the methodology section, illustrating the variation in environmental conditions affecting heat impacts.

 “Units 1 to 8 had a similar interior characteristics. The units contain a built-in cupboard and closet, a stove, a fridge, TV stand, couch/chairs and a bed. Unit 9, which was the salon had a wall-to-wall table with 7 chairs, a cabinet where they store their tools, and some tools were, displayed on the table. The office contained a table, four chairs and a small free-standing cabinet.”

 4. Besides that, this study incorporated a questionnaire survey and physical measurements. It would be nice if the author could associate the survey findings with the results from the physical measurement, where there is no discussion about it.

**Response:** We thank the reviewer for their insightful feedback and suggestion to link physical measurements to survey findings. We appreciate their thoroughness in reviewing our work. Unfortunately, the request cannot be fulfilled due to logistical constraints as this data cannot be linked.

 5. Under the results section, apparent temperature findings mostly presented the data and lacked critical discussion, which reflected the significant AT difference between the units, e.g., paragraphs 3 and 4 in the results section. More references to support the judgment and the input for different environmental conditions may be helpful in this case.

 Response: You rightly pointed out that paragraphs 3 and 4 in the results section primarily present the data without critical discussion. However, It is a common practice to maintain a clear distinction between the results and discussion sections to enhance the clarity and organization of the manuscript. The results section is intended to present the findings without interpretation, allowing readers to objectively evaluate the data. On the other hand, the discussion section serves the purpose of interpreting the results, comparing them with existing literature, and providing a broader context for the study.

 By adhering to this separation, we aim to ensure a logical flow and readability of the manuscript. Therefore, we propose that the critical discussion of the apparent temperature findings, along with additional references to support the judgment and insights on different environmental conditions, be appropriately addressed in the dedicated discussion section. We are committed to enhancing the manuscript based on your valuable suggestions and we have added additional insights in the discussion section.

  

- **Is the work clearly and accurately presented and does it cite the current literature?**

Yes

- **Is the study design appropriate and is the work technically sound?**

Yes

- **Are sufficient details of methods and analysis provided to allow replication by others?**

Yes

- **If applicable, is the statistical analysis and its interpretation appropriate?**

Partly

- **Are all the source data underlying the results available to ensure full reproducibility?**

Partly

- **Are the conclusions drawn adequately supported by the results?**

Yes
